# Interventions are needed to support patient–provider decision-making for DCIS: a scoping review

**DOI:** 10.1007/s10549-017-4613-x

**Published:** 2017-12-23

**Authors:** Claire Kim, Laurel Liang, Frances C. Wright, Nicole J. Look Hong, Gary Groot, Lucy Helyer, Pamela Meiers, May Lynn Quan, Robin Urquhart, Rebecca Warburton, Anna R. Gagliardi

**Affiliations:** 1 University Health Network, Toronto, Canada; 2 Sunnybrook Health Sciences Centre, Toronto, Canada; 3 University of Saskatchewan, Saskatoon, Canada; 4 Dalhousie University, Halifax, Canada; 5 University of Calgary, Calgary, Canada; 6 University of British Columbia, Vancouver, Canada

**Keywords:** Ductal carcinoma in situ, Communication, Decision-making, Supportive care

## Abstract

**Purpose:**

Prognostic and treatment uncertainty make ductal carcinoma in situ (DCIS) complex to manage. The purpose of this study was to describe research that evaluated DCIS communication experiences, needs and interventions among DCIS patients or physicians.

**Methods:**

MEDLINE, EMBASE, CINAHL and The Cochrane Library were searched from inception to February 2017. English language studies that evaluated patient or physician DCIS needs, experiences or behavioural interventions were eligible. Screening and data extraction were done in duplicate. Summary statistics were used to describe study characteristics and findings.

**Results:**

A total of 51 studies published from 1997 to 2016 were eligible for review, with a peak of 8 articles in year 2010. Women with DCIS lacked knowledge about the condition and its prognosis, although care partners were more informed, desired more information and experienced decisional conflict. Many chose mastectomy or prophylactic mastectomy, often based on physician’s recommendation. Following treatment, women had anxiety and depression, often at levels similar to those with invasive breast cancer. Disparities were identified by education level, socioeconomic status, ethnicity and literacy. Physicians said that they had difficulty explaining DCIS and many referred to DCIS as cancer. Despite the challenges reported by patients and physicians, only two studies developed interventions designed to improve patient–physician discussion and decision-making.

**Conclusions:**

As most women with DCIS undergo extensive treatment, and many experience treatment-related complications, the paucity of research on PE to improve and support informed decision-making for DCIS is profound. Research is needed to improve patient and provider discussions and decision-making for DCIS management.

**Electronic supplementary material:**

The online version of this article (10.1007/s10549-017-4613-x) contains supplementary material, which is available to authorized users.

## Background

Approximately 15–25% of mammographically detected lesions are ductal carcinoma in situ (DCIS), an unintended consequence of screening mammography [1]. DCIS is a complex premalignant disease that includes a spectrum of abnormal cell types confined to the breast ducts with variable natural history and risk of progression and recurrence [1]. Approximately 20% of cases will progress to invasive ductal carcinoma so most women with DCIS will never develop invasive disease and have a favourable prognosis [1, 2]. The 20-year breast cancer-specific mortality is 3.3% (95% CI 3.0–3.6) [2]. Currently, there is no reliable way to determine which women with DCIS will develop invasive disease, although strategies for determining recurrence risk based on pathologic indicators are forthcoming [1]. DCIS may be more aggressive in women under the age of 50 compared with postmenopausal women [2]. However, until evidence emerges from ongoing trials [3] and guidelines are updated [4], it is not possible to triage women who may be able to achieve good oncologic outcomes with less invasive treatment. Consequently, as recommended by guidelines, most women undergo lumpectomy-alone, lumpectomy and adjuvant radiation and/or hormone therapy, or mastectomy, and may experience short- and long-term treatment-related complications [4, 5].

Women diagnosed with DCIS reported confusion about DCIS, treatment options (lumpectomy versus mastectomy, need for radiation therapy) along with possible complications, and their prognosis and, as a result, had poor health care experiences and adverse health outcomes [6–10]. Physicians also reported challenges in communicating with patients about DCIS: 22% of 296 physicians in the United Kingdom and 78% of 151 physicians in the United States said that it was difficult to explain DCIS and treatment options to patients [7, 8]. Patient engagement is defined as care that informs, educates, engages and activates patients consistent with their needs and values [11]. Research shows that patient engagement improves patient knowledge, relationship with providers, service experience and satisfaction, treatment compliance, health outcomes and cost-effective service delivery and use [12–14]. A Cochrane review showed that PE is more probable if strategies to support it are aimed not only at patients, but also at physicians who influence treatment choices [15].

Prognostic uncertainty and treatment options with associated potential complications make DCIS unique from, and more challenging than, management of invasive breast cancer for both patients and providers, resulting in detrimental experiences and outcomes for patients. PE is relevant in circumstances where there is limited evidence to support decision-making; two or more treatment options are suitable; or treatment outcomes are difficult to predict, or may be adverse, as is the case for DCIS [16]. Both patients and providers would benefit from interventions or tools such as education or decision aids that support patient engagement for DCIS. First, thorough insight is needed on DCIS communication experiences and interventions among patients and physicians. This would identify whether sufficient knowledge exists, or further research is needed to understand DCIS communication experiences and develop corresponding interventions. The purpose of this study was to describe the characteristics and findings of existing research that evaluated DCIS communication experiences, needs and interventions among patients or physicians.

## Methods

### Approach

A scoping review was chosen as the methodologic approach [14, 17, 18]. Similar in rigour to a traditional systematic review, the purpose of a scoping review is to gain an understanding of the extent of research on a given topic, reveal gaps in knowledge and identify issues warranting ongoing research [19]. A scoping review involves five steps: scoping the literature, searching, screening, data extraction and data analysis. The Preferred Reporting Items for Systematic Reviews and Meta-Analyses [20] criteria guided reporting of the methods and findings [14]. Data were publicly available so institutional review board approval was not needed. A protocol for this review was not registered.

### Scoping

The scoping process involved becoming familiar with the literature on this topic using few high-level search terms so as not to eliminate any relevant concepts. A preliminary search was conducted in MEDLINE using Medical Subject Headings including, but not limited to, (ductal carcinoma in situ) and (patient education as topic or patient-centered care). CK and ARG screened titles and abstracts of the preliminary search results, which were used to plan a more comprehensive search strategy and to generate eligibility criteria based on the PICO (population, intervention, comparisons, outcomes) framework. The PICO framework is commonly used in systematic reviews to thoroughly address all relevant eligibility criteria such that subsequent searching and screening are optimized. All members of the research team, composed of health services researchers and general surgeons who care for cancer patients, reviewed eligibility criteria and provided feedback.

*Populations* referred to both patients and health care professionals. Patients included those diagnosed with DCIS. Health care professionals were practising physicians who manage patients with DCIS including general surgeons, and surgical, radiation and medical oncologists because they are the individuals who discuss diagnosis and treatment options with patients. *Interventions* included any policy, programme or single- or multi-faceted strategy implemented to promote awareness, understanding and discussion about DCIS. With respect to *comparisons*, studies were eligible if they explored or evaluated the following aspects of DCIS: understanding of the disease; views about the disease; communication about the disease; experiences and psychosocial outcomes of undergoing treatment for DCIS; determinants or factors influencing DCIS understanding, views, communication, experiences or choice; or behavioural interventions to support or improve any of these functions by comparing patients or providers with and without exposure to interventions, or before or after exposure to interventions, or receiving different types of interventions. *Outcomes* were those reported in eligible studies and included but were not limited to awareness, understanding, communication, experiences or impacts of DCIS, or determinants or factors influencing any of these functions, or the impact of behavioural interventions implemented to support or improve any of these functions. Eligible study designs included English language qualitative (interviews, focus groups, qualitative case studies), quantitative (questionnaires, randomized controlled trials, time series, before/after studies, prospective or retrospective cohort studies, case–control studies) or mixed methods studies. Systematic reviews were not eligible, but their references and those of all eligible studies were screened to identify additional eligible primary studies.

### Searching

The search strategy was developed in conjunction with a medical librarian and complied with the Peer Review of Electronic Search Strategy reporting guidelines (Table 1) [21]. MEDLINE, EMBASE, CINAHL, and the Cochrane Library were searched on February 16, 2017 from inception to that date.

**Table 1:** Search strategy

#	Searches	Results
1	Carcinoma, Intraductal, Noninfiltrating/	8623
2	CARCINOMA, DUCTAL/ [used 1963-93, use CARCINOMA, INTRADUCTAL, NONINFILTRATING to search CARCINOMA, DUCTAL 1966-93]	1187
3	limit 2 to yr=“1902 - 1965”	124
4	dcis.mp.	3612
5	ductal carcinoma* in situ.mp.	5237
6	(carcinoma* adj4 intraductal).mp.	9543
7	Paget’s Disease, Mammary/	698
8	1 or 3 or 4 or 5 or 6 or 7	13041
9	Patient Education as Topic/	77452
10	patient education handout/	4531
11	(patient* adj4 educat*).mp.	100404
12	Comprehension/	10834
13	(readable or readability).mp.	2895
14	exp Learning/	328881
15	(patient* adj4 learn*).mp.	5677
16	(patient* adj4 know*).mp.	39386
17	(patient* adj4 understand*).mp.	14038
18	(patient* adj4 (comprehend* or comprehension*)).mp.	950
19	exp Informed Consent/	38029
20	informed.mp.	87866
21	(patient* adj4 communicat*).mp.	16285
22	exp Communications Media/	273700
23	(information adj4 needs).mp.	5233
24	(information adj4 obtain*).mp.	42149
25	((apply or applie? or applying or application?) adj4 information).mp.	5881
26	(patient* adj4 (explain??? or explanation?)).mp.	7471
27	(educat* adj4 (barrier* or facilitat*)).mp.	2810
28	(learn* adj4 (barrier* or facilitat* or challeng*)).mp.	5599
29	(know* adj4 (barrier* or facilitat* or challeng*)).mp.	6047
30	(understand* adj4 (barrier* or facilitat* or challeng*)).mp.	10614
31	((comprehend* or comprehension*) adj4 (barrier* or facilitat* or challeng*)).mp.	354
32	(communicat* adj4 (barrier* or facilitat* or challeng*)).mp.	10641
33	(information adj4 (barrier* or facilitat* or challeng*)).mp.	5390
34	((knowledge or information) adj4 access*).mp.	15943
35	((knowledge or information) adj4 broker*).mp.	199
36	((knowledge or information) adj4 spread*).mp.	1128
37	((knowledge or information) adj4 flow???).mp.	5329
38	((knowledge or information) adj4 collect*).mp.	23574
39	(translat* adj4 (information or knowledge)).mp.	4584
40	((knowledge or information) adj4 exchang*).mp.	6091
41	((information or knowledge) adj4 (acquisition or acquir*)).mp.	10596
42	((information or knowledge) adj4 gain???).mp.	13039
43	exp Communication/ [includes communication barriers, health communication etc.]	420493
44	misunderstand*.mp.	3836
45	miscommunicat*.mp.	489
46	mistaught.mp.	1
47	misinform*.mp.	1905
48	(communicat* adj4 (fail* or error*)).mp.	1408
49	(understand* adj4 (fail* or error*)).mp.	1986
50	misunderstood.mp.	1737
51	(incomprehend* or incomprehension*).mp.	48
52	confus???.mp.	43867
53	uninform*.mp.	2047
54	(knowledge* adj4 (fail* or error*)).mp.	1165
55	(information* adj4 (fail* or error*)).mp.	2531
56	((knowledge or information) adj4 lack???).mp.	21650
57	(communicat* adj4 lack???).mp.	1521
58	(understand* adj4 lack???).mp.	4506
59	((explanation* or explain*) adj4 lack???).mp.	4180
60	((teach* or taught) adj4 lack???).mp.	403
61	(educat* adj4 lack???).mp.	2559
62	(knowledge adj4 gap?).mp.	9651
63	(information adj4 gap?).mp.	1323
64	(communication adj4 gap?).mp.	3720
65	(understanding adj4 gap?).mp.	2150
66	(understanding adj4 gain???).mp.	10192
67	(knowledge adj4 (inaccura* or incomplete* or incorrect*)).mp.	1046
68	(information adj4 (inaccura* or incomplete* or incorrect*)).mp.	2399
69	((explanation* or explain*) adj4 (inaccura* or incomplete* or incorrect*)).mp.	586
70	((teach* or taught) adj4 (inaccura* or incomplete* or incorrect*)).mp.	56
71	(communicat* adj4 (inaccura* or incomplete* or incorrect*)).mp.	112
72	(understand* adj4 (inaccura* or incomplete* or incorrect*)).mp.	1382
73	((teach* or taught) adj4 (barrier* or facilitat*)).mp.	935
74	(patient* adj4 (teach* or taught)).mp.	6701
75	(learn* adj4 (fail* or error* or lack??? or gap? or incomplete* or inaccura* or incorrect*)).mp.	3492
76	exp Patient Satisfaction/	73809
77	(patient* adj4 satisf*).mp.	97266
78	(patient* adj4 experienc*).mp.	106283
79	(patient* adj4 prefer*).mp.	23059
80	“illness experience?”.mp.	1134
81	“diagnos* experience?”.mp.	307
82	“prognos* experience?”.mp.	14
83	“treatment* experience?”.mp.	2765
84	“follow-up experience?”.mp.	182
85	“survivorship experience?”.mp.	57
86	“experience of illness”.mp.	491
87	“experience of diagnos*”.mp.	928
88	“experience of prognos*”.mp.	68
89	“experience of treatment*”.mp.	1967
90	“experience of follow-up”.mp.	177
91	“experience of survivorship”.mp.	12
92	CONSUMER SATISFACTION/ [use to search PATIENT SATISFACTION 1982-91]	19040
93	exp Public Relations/	109728
94	(positive adj4 experience*).mp.	6949
95	(negative adj4 experience*).mp.	5360
96	(good adj4 experience*).mp.	2073
97	(bad adj4 experience*).mp.	338
98	(respect* adj4 interact*).mp.	4643
99	(respect* adj4 treat*).mp.	33437
100	(respect* adj4 conversat*).mp.	42
101	disrespect*.mp.	505
102	(good adj4 communicat*).mp.	1988
103	(poor adj4 communicat*).mp.	1576
104	(respect* adj4 communicat*).mp.	730
105	(shar* adj4 decision).mp.	4117
106	(enough adj4 information*).mp.	1736
107	(enough adj4 communicat*).mp.	63
108	(sufficien* adj4 information*).mp.	4632
109	(sufficien* adj4 communicat*).mp.	187
110	(insufficien* adj4 information*).mp.	2211
111	(insufficien* adj4 communicat*).mp.	238
112	listening.mp.	12362
113	Decision Making/	79019
114	exp Choice Behavior/	46792
115	(decision* or decide* or deciding).mp.	344149
116	(choice* or choose* or chose*).mp.	375015
117	exp patient centered care/	14724
118	(patient* adj2 (centered or centred)).mp.	21245
119	exp Professional-Patient Relations/	130244
120	(relations* adj4 (physician* or doctor* or professional*) adj4 patient*).mp.	102931
121	health literacy/	3159
122	health literac*.mp.	4914
123	health literate.mp.	89
124	exp Attitude to Health/	349676
125	(health adj4 (knowledg* or attitude*)).mp.	255723
126	Practice Patterns, Physicians’/	48762
127	practice pattern*.mp.	55115
128	pattern* of practice.mp.	485
129	Professional Practice/	16018
130	exp Consumer Participation/	36274
131	(participat* adj4 (patient* or consumer* or client*)).mp.	42306
132	decision support techniques/	15967
133	Decision Support Systems, Clinical/	6197
134	Patient Care Management/	2811
135	exp Patient Care Planning/	58229
136	disease management/	26772
137	(care adj2 (manag* or plan*)).mp.	89775
138	“Quality of Health Care”/	64024
139	Quality Improvement/	12994
140	exp Quality Assurance, Health Care/	288987
141	(quality adj4 care).mp.	157694
142	(quality adj4 improv*).mp.	110356
143	(quality adj4 (high or low)).mp.	57255
144	(quality adj4 increas*).mp.	12900
145	(quality adj4 (good or poor or bad)).mp.	28967
146	(quality adj4 (better or worse*)).mp.	13421
147	(quality adj4 (assur* or ensur*)).mp.	70255
148	or/9-147	2984559
149	8 and 148	1611
150	remove duplicates from 149	1567
151	exp animals/ not (exp animals/ and exp humans/)	4319036
152	150 not 151	1562
153	limit 152 to (“all adult (19 plus years)” or “young adult (19 to 24 years)” or “adult (19 to 44 years)” or “young adult and adult (19-24 and 19-44)” or “middle age (45 to 64 years)” or “middle aged (45 plus years)” or “all aged (65 and over)” or “aged (80 and over)”)	1031
154	limit 152 to (“all infant (birth to 23 months)” or “all child (0 to 18 years)” or “newborn infant (birth to 1 month)” or “infant (1 to 23 months)” or “preschool child (2 to 5 years)” or “child (6 to 12 years)” or “adolescent (13 to 18 years)”)	54
155	152 not 154	1508
156	153 or 155	1559
157	limit 156 to female	1458
158	(wom#n or female?).mp.	7645637
159	156 and 158	1469
160	157 or 159	1469
161	limit 160 to english language	1343
162	160 not 161	126

### Screening

To prepare for screening, CK and ARG independently screened the title and abstract of the first 25 search results, then compared and discussed discrepancies and how to interpret and apply the eligibility criteria. CK, LL and ARG screened titles and abstracts according to specified PICO-based eligibility criteria. Criteria for ineligible studies were generated prospectively with screening. Studies were not eligible if they primarily involved health care providers other than practising physicians who discuss diagnosis and treatment with patients (nurses or allied health care professionals including but not limited to physiotherapists, speech therapists, occupational therapists, social workers, pharmacists; or trainee physicians such as interns, residents or fellows; studies were included if at least half the participants were practising physicians); examined the clinical effectiveness of DCIS treatment options; or were in the form of protocols, editorials, commentaries, letters, news items, meeting abstracts or proceedings. All items selected by at least one reviewer were retrieved.

### Data extraction

A data extraction form was developed to collect information on study characteristics including author, publication year, country, study objective, research design, participants and findings. If an intervention was employed, data were also extracted on content (information/knowledge conveyed), format (mode of delivery, single- or multi-faceted), timing (duration, frequency), participants (number, type, setting) and personnel who delivered the intervention according to the Workgroup for Intervention Development and Evaluation Research [22] reporting standards for behavioural interventions [14, 17, 18]. To pilot data extraction, CK, LL and ARG independently extracted data from the same three articles, and compared and discussed findings to refine the data extraction form. CK and LL extracted data from all articles, which were independently checked by ARG.

### Data analysis

Summary statistics were used to report the number of studies published per year, by type of cancer, in different countries and according to study design. Study findings were reported narratively. Methodological quality of included studies was not assessed as this is not customary for a scoping review.

## Results

### Search results

**Fig. 1:**
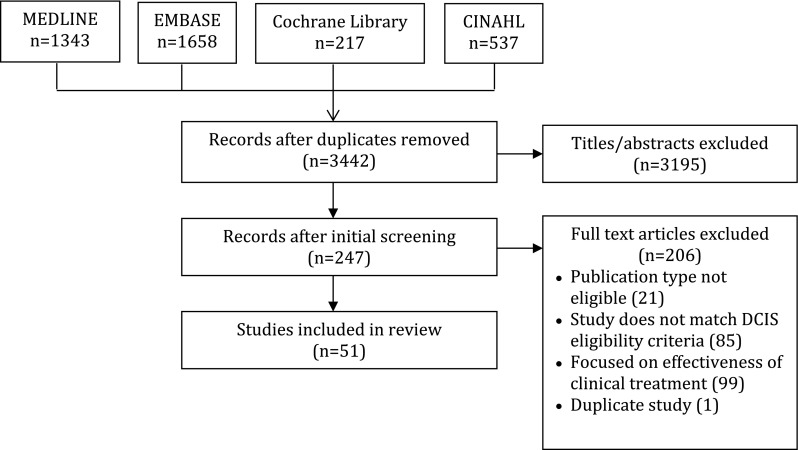
PRISMA diagram

A total of 3753 studies were identified by searches, of which 3442 were unique items, and 3195 were excluded based on screening of titles and abstracts. Among 247 full-text articles that were screened, 206 were excluded because they focused on effectiveness of clinical treatment (99), studies did not match DCIS eligibility criteria (85), the publication type was not eligible (21), or duplicate (1). Of 27 systematic reviews identified through screening, two were relevant and 10 additional eligible primary studies were identified among their references. A total of 51 studies were eligible for review (Fig. 1). Data extracted from included studies are available in Table 2 and discussed here [23–73]. Themes that emerged from the included studies are summarized in Table 2.

**Table 2:** Themes that emerged from included studies

Domain (*n* studies)	Themes	Studies
Knowledge about DCIS and its prognosis [14]	Little awareness or knowledge about DCIS	[23, 41, 46, 57, 63]
	Failure to distinguish DCIS from invasive breast cancer	[41, 42, 45, 63, 64, 66, 73]
	Inaccurate perception of risk for invasiveness/recurrence	[35, 36, 49, 50]
	Influenced by socioeconomic status, race	[35, 36]
Communication and decision-making [28]	Sources of information (in no particular order): surgeons, breast cancer nurses, Internet, books, leaflets, medical journals, cancer charities	[58, 63, 71]
	Not satisfied with information about DCIS provided to them	[70, 72]
	High level of decisional conflict	[42, 46, 59]
	Decisions highly influenced by physician recommendation, patient age, race, patient concern about recurrence	[24, 25, 28, 30, 33, 37, 38, 44, 55, 60, 65, 68]
	Informed or shared decision-making influenced by socioeconomic status	[30, 33, 38, 44]
	Challenges faced by physicians were uncertainty about appropriate treatment and explaining DCIS to patients	[54, 56]
	Physicians referred to DCIS as abnormal cells, early form of cancer, cancer, cancerous or malignant cells and non-invasive cancer	[25, 26, 29, 39, 42, 56, 58, 67]
Psychosocial impact of DCIS [19]	Range of emotions from calm acceptance and relief that disease caught early through to shock and distress	[39, 46, 47, 52, 73]
	Following treatment women experienced worsened body image, lower quality of life, poor relationships with others, decreased sexual desire or activity, tension, anxiety, loneliness and depression	[31, 48, 52, 58, 62, 70, 72]
	Some women reported high degree of social support, little impact on sexual function or quality of life, or little strain on interpersonal relationships compared with women who had invasive breast cancer	[31, 34, 40, 51, 53, 58, 64, 66]
	Influenced by socioeconomic status, no partner and age	[32]
Interventions to support DCIS communication or decision-making [2]	Women with DCIS and physician thought that communication or decisions aids would help patients understand DCIS, and its treatment and prognosis	[27, 43]

### Study characteristics

**Fig. 2:**
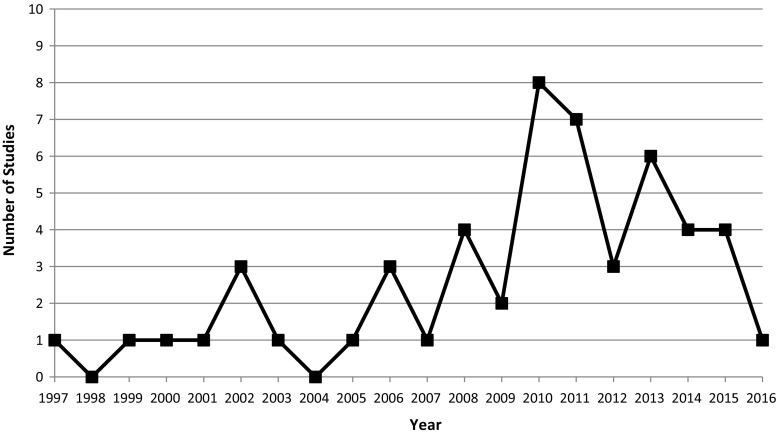
Studies published per year

The number of studies generally increased from 1997 to 2016, peaking at 8 articles in year 2010 (Fig. 2). Studies were conducted in the United States (28), United Kingdom (9), Australia (6), Canada (3), Italy (1), Netherlands (1), Sweden (1), Switzerland (1) and Tasmania (1). With respect to research design, most studies involved cross-sectional questionnaires (21, 41.2%), followed by qualitative interviews or focus groups (19, 37.3%), single cohorts (4, 7.8%), mixed methods (5, 9.8%) and comparative cohorts (2, 3.9%).

### Knowledge about DCIS and prognosis (*n* = 14 studies)

Four studies found that none or few women had heard of DCIS prior to diagnosis [41, 46, 57, 63]. Another four studies found that women with DCIS were unsure of whether or not their disease was invasive [41, 42, 63, 73]. Several studies found that women overestimated their risk from DCIS [49] and worry about dying from breast disease was significantly associated with thinking that DCIS could metastasize [42]. A study of 181 women with DCIS found that participants perceived a moderate risk of DCIS spreading in the body (24%), developing DCIS again within 5 years (32%), developing DCIS again within their lifetime (43%), developing invasive breast cancer within 5 years (27%) and developing invasive cancer within their lifetime (38%) [36]. Women who were financially comfortable or at least college graduates were less likely to perceive that DCIS could spread, or that the risk of DCIS was moderate or large [36]. Fifteen percent of DCIS patients in one study reported their recurrence risk to be > 50% [50]. Another study found that, although 41.0% of DCIS patients were aware that their disease was not life threatening, 15.0% of patients reported their recurrence risk to be greater than 50.0% [35]. English-speaking whites were more likely to know that DCIS is not life-threatening compared with Latina women (OR 95% CI 90.6, 0.4–0.9 and 0.5, 0.3–0.9, respectively) [35]. Two studies found that partners of patients were more likely to know about risk of recurrence compared with the patients (*p* = 0.003) [23]. Three studies found that DCIS and invasive breast cancer patients had comparable risk perceptions concerning the risk of recurrence [45, 64, 66], and two studies found similar risk perceptions of dying of their disease [64, 66].

### Communication and decision-making (*n* = 28 studies)

Women reported that surgeons [58, 71] and breast cancer nurses [39] were important providers of information about DCIS. Two studies found that patients were not satisfied with the information they received about DCIS [70, 72]. Women acquired information from various sources including the Internet [63, 71], books, leaflets, medical journals, cancer charities, and health professionals [63]. Three studies found that women diagnosed with DCIS expressed high decisional conflict regarding treatment choice [42, 46, 59]. Patients said that their surgeon discussed both mastectomy and breast-conserving surgery, and those who chose mastectomy were influenced by concern of recurrence [65]. In an American study comparing Whites to Latinas, discussion of therapy or treatment decisions was less likely with Spanish-speaking Latinas. This group was also less affluent, less educated, had lower rates of employment, and were less likely to be privately insured [38] or report making decisions together with their physicians [30, 33, 44]. A study of treatment decision-making among Chinese-Canadian women found that they wanted to get rid of breast cancer once and for all and were influenced by physician recommendations [60]. Patients with DCIS who opted for mastectomy were more likely to be younger and have higher grade tumours [25, 37, 44] compared with those undergoing breast-conserving surgery. One study found that younger age was associated with mastectomy [44, 65], while another found that breast-conserving surgery was more likely among younger patients [68]. Those who chose contralateral prophylactic mastectomy were younger or married [24, 28, 55], or white race or having the presence of lobular carcinoma in situ [55].

According to healthcare professionals, the most common challenge for DCIS patients was “understanding the condition” [56]. Physicians said that the greatest challenge they faced pertained to uncertainty about appropriate treatment [54]. One study found that 51.4% of the health care professionals surveyed found DCIS more difficult to explain to patients than invasive cancer (only 9% found DCIS easier to explain) [56]. DCIS was described by physicians using a variety of terms including abnormal cells [26, 56], pre-cancer or pre-invasive breast cancer cells [25, 26, 39, 56], abnormal cells in the milk ducts [67], earliest possible form of breast cancer [39], not breast cancer as we commonly think of breast cancer [42], cancer, cancerous cells, malignant cells, changes [56], and non-invasive cancer [58]. One study found that physicians most preferred DCIS defined as “abnormal cells in the milk ducts that had not spread to other breast tissues and which did not need urgent treatment” and least preferred the definition, “the earliest possible form of breast cancer and is non-invasive” [29].

### Psychosocial impact of DCIS (*n* = 19 studies)

In some cases, women accepted their diagnosis calmly [73] and others were relieved that their disease was caught early [39]. Two studies interviewed women with DCIS and found common themes to be that it was a challenge to body integrity and identity [52] and gave a feeling of ongoing risk [39], though the possibility of reconstruction was of some comfort [52]. Women who needed a mastectomy were often very shocked and upset [46]. Women who underwent an immediate reconstruction for DCIS reported greater overall body image distress than breast-conserving surgery patients (*p* = 0.001) and marginally higher levels than those who underwent mastectomy without reconstruction (*p* = 0.055) [47]. Another study found that after treatment of DCIS, some women reported perceptions of a worsened body image (16%), tension (46%), nervousness (48%), loneliness (29%), anxiousness (59%) and depression (41%) [72]. Two studies found that, over time, anxiety and depression declined [47, 58].

Four studies examined social outcomes of DCIS diagnosis and found that women reported a high degree of social support [31, 58] and women with DCIS reported less withdrawal from close family/friends (5% vs. 11%, *p* = 0.08) and strain on interpersonal relationships (0% vs. 6%, *p* = 0.02) compared with women with early invasive breast cancer [66]. However, one study reported that DCIS negatively affected patients’ relationships with others [52].

Five studies examined the effects of DCIS on sexual function. In two studies, women with DCIS appeared to have very similar sexual function as women without DCIS [34, 51] and, in one study, women with DCIS experienced a less negative effect on their sex life compared with women with invasive breast cancer (*p* = 0.03) [64]. Half of the DCIS patients in one study reported decreased interest in sex and decreased sexual activity [70], and another 5% of patients in another study reported some limitations in sexuality, interference with sexual desire and modifications during intercourse [72].

When compared with women without breast disease, one study found that women with DCIS had statistically greater declines in quality of life [62], whereas another study found that women treated for DCIS had a similarly satisfactory quality of life [53]. Two studies found that patients with DCIS experienced better quality of life compared with invasive breast cancer patients [40, 64], while two other studies reported that DCIS patients and invasive breast cancer patients experienced similar levels of distress [31, 48]. Factors associated with lower quality of life were younger age, no partner and lower income [32].

### Interventions to support communication or decision-making (*n* = 2 studies)

Two studies investigated interventions to facilitate DCIS communication and decision-making [27, 43]. In one study that developed a communication aid, DCIS patients and health care professionals felt that it would help women to understand their diagnosis, treatment and prognosis [43]. In another study, physicians said that they would be interested in using a web-based decision aid (http://www.onlinedecision.org/) that included various educational materials such as a lay language description of treatment options, outcomes data and communication support [27].

## Discussion

A considerable proportion of mammographically detected lesions are DCIS, yet little research spanning 1997–2016 investigated the treatment decision-making experiences of patients or providers. Most women had little knowledge of DCIS and inaccurate perceptions of associated risks and prognosis. Physician recommendations and patient factors informed treatment decision-making and, as a result, women experienced high decisional conflict and were not satisfied with information provided to them. Many chose mastectomy, an acceptable option for women with a large area of disease or the desire to avoid radiation, or prophylactic mastectomy. Following treatment, women reported anxiety and depression, often at levels similar to those with invasive breast cancer. Disparities were identified by education level, socioeconomic status, ethnicity and literacy. Physicians agreed that patients did not understand the condition, but said that they had difficulty explaining it and many referred to it as cancer. Despite the challenges reported by patients and physicians, no studies evaluated decision aids or other policies, programmes or strategies to promote awareness, understanding and discussion about DCIS; only two studies explored patient or provider interest in communication or decision aids. Given the fact that most women with DCIS undergo extensive treatment, which is the present standard [4, 5], and many experience treatment-related clinical and psychosocial sequelae, the paucity of research on PE to improve and support patient–provider communication and informed decision-making for DCIS is profound.

The findings of our review also emerged in other research. For example, researchers have explored the influence of terminology on subsequent treatment decision-making. In one study of 269 women, those first exposed to the term “abnormal cells” then later “pre-invasive breast cancer cells” were more likely to feel concern and change their management preference to treatment compared to women exposed first to the term “pre-invasive breast cancer cells” and then “abnormal cells,” however, there was no significant difference in treatment preferences between the two groups (*p* = 0.23) [26]. In another study, 26 women who were interviewed said that they would feel concern regardless of the term used to describe DCIS but preferred the term abnormal cells over other terms such as carcinoma, and expressed interest in active surveillance over immediate treatment provided monitoring was very frequent [74]. Interviews with 29 early-stage breast cancer patients who underwent unilateral or contralateral prophylactic mastectomy revealed that fear translated into an overestimated risk of recurrence, contralateral breast cancer and death despite advice from surgeons [75]. Rutherford et al. reviewed the literature on treatment decision-making in DCIS [76]. While they identified themes similar to those that emerged in our study, they searched from database inception to November 2015 and included 22 articles, while we searched from database inception to February 2017 and included 51 articles; thus, our review is more current. Our review is also more comprehensive because we searched for patient or provider interventions that support communication about DCIS while Rutherford did not; consequently, a unique finding of this study was the lack of interventions aimed at patients or providers to support communication and decision-making for DCIS.

Active surveillance is a new option for managing select cancer patients to reduce potential over-treatment and the associated sequelae that can impact health and health-related quality of life [77]. It avoids or postpones definitive DCIS treatment until there is evidence from periodic observation or testing that a patient is at greater risk of or has disease progression [78]. Active surveillance has become a standard option for managing prostate cancer, and trials are currently underway to establish the clinical, molecular and psychosocial outcomes of active surveillance for DCIS [79–81]. While many are striving to improve the clinical management of DCIS, trial results are uncertain and may not be available for many years. Even if active surveillance becomes a management option for DCIS, the confusion among women caused by a diagnosis of DCIS, and the dilemma experienced by physicians in recommending treatment for a potentially benign condition remains. This review underscored the impact of DCIS on psychosocial issues and health-related quality of life, and revealed an imperative for interventions to address the needs of women diagnosed with DCIS. Engaging patients in their own care improves patient, provider and system-level outcomes [12–14]. A framework of person-centred cancer care stipulates the interdependence of six domains that must be addressed to provide better support to DCIS patients: fostering the patient–provider relationship, exchanging information, responding to patient emotions, managing uncertainty, making decisions and enabling patient self-management [82]. At the same time, physicians must be provided with education and tools that enable them to address these domains.

Hence, further research is needed to develop resources or tools that support communication, and informed or shared decision-making for DCIS. A Cochrane systematic review of 105 studies including 31,043 participants showed that decision aids improved knowledge, accurate risk perception and values-congruent choices when used either within or in preparation for consultation [83]. However, other research shows that awareness and use of decision aids among physicians may be limited [84, 85]. Given that, in this review, physicians influenced treatment choices but referred to DCIS as cancer and said that DCIS was challenging to describe to patients, physicians may require training to more accurately and better engage patients in discussions about DCIS. Further research is needed to understand the implementation and impact of decision aids, particularly in the context of patients who may be disadvantaged by factors such as low literacy. A range of types of tools other than decision aids can be just as effective and should also be studied in the context of DCIS. For example, print (brochures, booklets, variety of print material, list of websites) or electronic (video, computer program, website) material offered directly before, during or upon conclusion of consultations by health care professionals, health educators, researchers or volunteers improved patient knowledge, communication, decision-making and health care behaviour [86]. Also, increasingly patients or family members are being engaged in improving the quality of health services, often as patient navigators—perhaps women who were treated for DCIS could function as coaches to provide supportive care for women newly diagnosed with the condition [16].

A few issues may limit the interpretation and use of these findings. Although we searched the most relevant databases of medical literature with a search that complied with standards [21], and employed rigorous searching and screening processes, we may not have identified all relevant studies. We did not search the grey literature, referring to informally published resources such as organizational reports or the content of web sites, because most empirical research would be found in indexed databases, and because there are no standards for doing so, and grey information may be at high risk of bias [87, 88]. Publication bias, or the tendency for journals to publish studies with positive results or surveys with high response rates, may have influenced the number and type of studies that were retrieved. Given the wide range of processes and outcomes measured and reported across included studies, it was not possible to pool findings.

Despite these limitations, the purpose of this study was to assess the state of research on DCIS communication and decision-making to serve as a springboard for ongoing research in this area. In summary, this review summarized two decades of accumulated research on the challenges associated with DCIS diagnosis and management faced by women and physicians. This contrasts starkly with the absence of approaches, strategies or tools available to support communication and decision-making about DCIS, yet reveals opportunities by which the quality of care can be improved.

## Electronic supplementary material

Below is the link to the electronic supplementary material.

Supplementary material 1 (DOCX 81 kb)

## Abbreviations

DCIS: Ductal carcinoma in situ
