# Supplementary material for: Interventions are needed to support patient–provider decision-making for DCIS: a scoping review
Source: Breast Cancer Res Treat. 2017 Dec 23;168(3):579–92. doi: 10.1007/s10549-017-4613-x (PMC5842253; doi:10.1007/s10549-017-4613-x)
Supplement: Supplementary file 1 — Supplementary material 1 (DOCX 81 kb) [file 10549_2017_4613_MOESM1_ESM.docx]

Table S1. Data extracted from included studies

| Study | Research Design | Objective | Participants | Results |
| --- | --- | --- | --- | --- |
| Janz [23]  2016  USA | Questionnaire | To describe racial/ethnic differences and clinical/treatment correlates of worry about recurrence and examine modifiable factors in the health care experience to reduce worry among breast cancer survivors, partners, and pairs. | The study population included breast cancer survivors and their partners. Eligible participants were women 20-79 years old, diagnosed with DCIS or invasive breast cancer from June 2005 through February 2007.  The final sample included 510 pairs of survivors and their respective partners. 125 (24.5%) had DCIS. | - Partners were significantly more likely to report receiving enough information about the risk of recurrence than survivors (71.1 vs. 64.7%, p=0.003), but less likely to report receiving sufficient emotional support from providers (45.7 vs. 75.0%, p<0.001). - Partners were significantly more likely to report worry about recurrence at 4 years after diagnosis than the survivors (42.3 vs. 27.2%, p<0.001). - For survivors, the likelihood of worry was significantly higher among those with younger age, lower education levels, or worse health status. Latinas with low acculturation were most likely to express worry (50%), compared with 36.5% for Latinas with high acculturation, 27.1% for Whites, and 14.0% for Blacks. - For partners, the likelihood of worry was significantly more likely among those who were less educated or had worse health themselves. Similar to survivors, partners worry differed among racial/ethnical groups. Latino partners reported the highest percentages of worry, with 67.2% and 66.7% for low and high acculturation groups, respectively, compared with 44.3% for Whites and 27.1% for Black partners. - Partners of survivors who received chemotherapy reported more worry than the partners of those who did not (56.3 vs 40.0%, p<0.001). - The percentages of reported worry were consistently higher among the partners than the survivors. - Survivors and pairs who received sufficient emotional support form providers were less likely to worry than those who did not receive such support. - 25 (22.9%) DCIS patients worried about recurrence and 45 (41.3%) DCIS patient partners worried about recurrence (no statistical significance for either). |
| Elsayegh [24]  2015  USA | Single cohort | To identify differences between BRCA-negative patients who underwent contralateral prophylactic mastectomy and those who did not. | 100 patients who had a diagnosis of DCIS between 2005 and 2013 who had negative *BRCA* genetic test results.  31 elected contralateral prophylactic mastectomy and 69 did not. | - 67 patients underwent mastectomy for the affected breast and 31 underwent only breast conservation surgery, (unknown for 2 patients). - Married patients were more likely to elect contralateral prophylactic mastectomy than those who were not (p=0.0235). - Patients with a first-degree relative with ovarian cancer were more likely to choose contralateral prophylactic mastectomy than those without such a relative (p=0.0278). - Race, ethnicity, age at diagnosis, education, stage, and biomarker status were not significantly associated with contralateral prophylactic mastectomy election. - Moreover, patients who had any relatives with ovarian cancer were more likely to choose contralateral prophylactic mastectomy than those without any relative with ovarian cancer (p=0.0425); however, when analyzed by family history of breast cancer (first-degree relative or total number), the differences were not statistically significant (p=0.2436 and 0.7198, respectively). - Patients receiving tamoxifen were less likely to choose contralateral prophylactic mastectomy than those who were not (p=0.0054). - Patients who underwent mastectomy were more likely to choose contralateral prophylactic mastectomy than those who did not undergo mastectomy of the affected breast (p<0.0001). - Finally, patients who underwent reconstruction were more likely to choose to have contralateral prophylactic mastectomy than those who did not (p<0.0001). |
| Mannu [25]  2015  UK | Mixed methods | To investigate whether the attitudes of surgeons towards different aspects of DCIS treatment varied by seniority of surgeon or by geographical region within the UK. | 80 surgeons responded to the survey.  57% of respondents were male and 89.8% of participants were aged 30-49 years and either consultant (39%) or specialist registrar (37%).  63% of participants were based in district general hospitals and 37% were based in university hospitals. | - The majority of respondents agreed with describing DCIS as "abnormal cells in the milk ducts" (57%) and "pre-cancer" (76%). - The use of "tumour" and "cancer," were more preferable to surgeons with higher caseloads (≥20 cases per month) than to those with lower caseloads. - Geographically, approximately 54.6% and 72% of surgeons in North of English and in Scotland, respectively, disagreed with the term "tumour" compared to only 40% of surgeons in South of England. Similar results were seen for the term "malignancy." - The majority of surgeons attributed a higher risk of progression to invasive breast cancer or death with a higher pathological grade of DCIS. - The majority (45%) of surgeons attributed the risk of low-grade DCIS progressing to invasive breast cancer at 10 years to be between 10 and 19%. 49% of surgeons felt that the risk of intermediate grade DCIS progressing to invasive breast cancer was 20-49%, and this risk rose to over 50% for high-grade (63%). - Approximately 29% of surgeons in Scotland believed that high-grade DCIS had >50% risk of invasive cancer at 10 years compared to 80% of surgeons in South of England and 59% in North of England. - Overall the majority of surgeons estimated this risk for low-grade DCIS to be 1% (39%), for intermediate grade DCIS to be 2-5% (43%), and for high-grade DCIS to be more than 6% (61%). - The vast majority of respondents felt that breast-conserving surgery alone is adequate for large (4cm) low-grade DCIS lesion. - A higher proportion of female surgeons suggested mastectomy with sentinel lymph node biopsy, as did a higher proportion of more junior surgeons. - Only 23% of respondents felt that premenopausal women who have had breast-conserving surgery with adequate margins for small low-grade DCIS require postoperative radiotherapy. - However, 71% did not agree with this and were equally represented across geographical regions and levels of seniority. - The majority of surgeons (65%) felt that women with DCIS should be followed up for 5 years after treatment, 25% felt that <5 years is adequate, and 10% felt that follow-up no longer than 5 years was required. These views did not vary by region, setting, or seniority. - Approximately 25% of surgeons felt that annual mammograms with repeat referral of any abnormality is detected was the ideal follow-up for these women. - An equal proportion (25%) felt that women should be seen by an oncologist for the first year and then annual mammograms thereafter. - Approximately 20% felt that women should be seen regularly during this period by the breast surgeon and only 2% felt that input of both breast surgeon and oncologist was required for the entire 5 years. |
| McCaffery [26]  2015  Australia | Comparative cohort | To investigate the effect of describing DCIS as 'abnormal cells' versus 'pre-invasive breast cancer cells' on women's concern and treatment preferences. | Community sample of Australian women (n=269) who spoke English as their main language at home. | - Women in both arms (arm A 'abnormal cells', arm B 'pre-invasive breast cancer cells') indicated high concern, but still indicated strong initial preferences for watchful waiting (64%). - Initial concern was high in both arms with 47% of women across arms indicating they would be 'extremely concerned' and 48% 'moderately concerned' following a diagnosis of the condition described (DCIS). There were no differences in initial concern or preferences by trial arm. - However, more women in arm A (abnormal cells' first term) indicated they would feel more concerned if given the alternative term ('pre-invasive breast cancer cells') compared to women in arm B who received the terms in the opposite order (67% arm A vs 52% arm B would feel more concerned, p=0.001). - More women in arm A also changed their preference towards treatment when the terminology was switched from 'abnormal cells' to 'pre-invasive breast cancer cells' compared to arm B. - In arm A, 18% of women changed their preference to treatment while only 6% changed to watchful waiting (p=0.008). In contrast, there were no significant changes in treatment preference in arm B when the terminology was switched (9% vs 8% change their stated preference). |
| Ozanne [27]  2015  USA | Intervention and interviews | To create a web-based decision aid, designed to provide tailored information about a patient's likelihood of benefits and harms of different treatment strategies for DCIS that is informed by a disease simulation model. | Physicians were recruited during Sept-Nov 2012 to participate in the testing of the decision aid prototype. Candidate participants were medical oncologists, radiation oncologists, or surgeons from both academic and community settings and geographically dispersed area. A patient advocate was also interviewed to provide feedback regarding both the decision aid prototype and the Web site. Four user tests were conducted to review the decision aid prototype (Phase I)-3 physician interviews and 1 patient advocate. | *Intervention features*   - The decision aid was designed to include the following core characteristics:   (1) provide tailored information about patients' survival and breast preservation stratified by age and risk of recurrence  (2) provide outcomes for each current standard treatment strategy for DCIS  (3) include the down-stream effects of each treatment strategy on treatment options at time of recurrence  (4) facilitate communication and shared decision making during consultations  (5) provide access via a web-based platform that can be updated and disseminated rapidly   - The development process included two phases. The first involved the development of a decision aid prototype, and the second phase involved the development of the Web site into which the decision aid was embedded.   *Intervention outcomes*   - There was virtual unanimity around recommendations regarding the functionality and clarity of the charts in the decision aid. During the development of the Web site with embedded decision aid (Phase II), 5 physicians were interviewed, in addition to the patient advocate. Using the results of the physician interviews, the Web site with embedded decision aid was developed to include the following components:   - Education-Lay language descriptions of treatment options available to women who have been diagnosed with DCIS; Referral to other appropriate resources that clinicians might access online to facilitate patient understanding of potential benefits and harms associated with the selection of a specific treatment.   - Patient inputs-Selection of which treatment options to be displayed; Flexibility to adjust the patient's age based on their physiologic age or "real age"; Expected risk of invasive cancer recurrence with lumpectomy and no radiation therapy; Expected risk of DCIS recurrence with lumpectomy and no radiation therapy.   - Outcome data-Likelihood of time-specific (10-year and lifetime) recurrence and survival outcomes for an individual patient; Information on the potential for breast conservation based on clinical indicators and choices of therapy available to a woman with DCIS; Projections of down-stream effects of each treatment option should cancer recur.   - Communication support-Graphical interface that presents numeric information in a clear manner; Key points for the clinician to discuss with the patient, designed to help facilitate the decision-making conversation. There are 3 main inputs for the decision aid: patient age, expected DCIS recurrence risk over 10 years, and expected recurrence risk for invasive breast cancer over 10 years with lumpectomy and no radiation therapy. The outputs of the decision aid include 10 year and lifetime recurrence risk for the ipsilateral breast, contralateral breast, and the likelihood of being alive in 10 years. - After review of the decision aid website, all physicians in the study found the decision aid useful and indicated they would be interested in using the tool with their patients with DCIS in their clinics. - Many of the physicians felt that having a visual presentation of the data was the most effective way to communicate with patients about their treatment options. - The majority also indicated that it was important to have a printout version of the information for patients to take home. |
| Elsayegh [28]  2014  USA | Single cohort | Evaluated factors associated with contralateral prophylactic mastectomy in patients with DCIS who underwent genetic counselling for *BRCA* testing. | 165 women with DCIS referred for genetic counselling between 2003 and 2011.  51.5% were ≤45 years old at diagnosis, 55.2% were non-Ashkenazi Jewish and 31% had a college education. | - Of 165 patients, 44 (27%) underwent contralateral prophylactic mastectomy. - Patients <45 years were more likely to elect contralateral prophylactic mastectomy (p=0.0098). - A *BRCA+* mutation was found in 17 patients (10.3%), and BRCA+ women were more likely to elect contralateral prophylactic mastectomy than BRCA- or untested women (p=0.0001). - Patients who had a family history of ovarian cancer (57.7%) were more likely to choose contralateral prophylactic mastectomy than those with no family history (p=0.0004). - Younger age, *BRCA+,* and an ovarian cancer family history remained significant in the multivariate model (p<0.008). |
| Fallowfield [29]  2014  UK | Questionnaire | To determine which descriptions of DCIS were deemed most accurate and appropriate. | 54 healthcare professionals attending the British Breast Group meeting in Feb 2013.  35 males and 14 females; 22 were surgeons, 12 oncologists, 7 radiologists, 7 scientists, 3 pathologists, 1 endocrinologist and 1 cancer geneticist. | - A majority (34/45; 63%) said they would be comfortable using the description that explained DCIS as abnormal cells in the milk ducts that had not spread into other breast tissues and which did not need urgent treatment as if it was breast cancer and this description was overall the most preferred (24/54; 44%). - Respondents (36/54; 66.6%) were least comfortable with the Macmillan description: DCIS is the earliest possible form of breast cancer and is non-invasive. - Although DCIS needs to be treated, it isn’t a life-threatening condition. Surgery is the most common treatment. This consensus was mirrored across all professional groups, with 70% (14/20) surgeons, 85% (6.7) radiologists, 66.7% (2/3) pathologists and 75% (9/12) of oncologists. |
| Lopez [30]  2014  USA | Qualitative study  (interviews) | To examine differences in treatment decision-making participation, satisfaction, and regret among Latinas and non-Latina whites with DCIS. | 745 (396 White, 349 Latinas) women diagnosed with DCIS between 2002 and 2005, aged ≥18 years, identified through the California Cancer Registry.  Mean age was 57 years and women over 60 comprised more than 1/3 of the sample. | - Among 745 participants, Spanish-speaking Latinas had the highest mean preference for involvement in decision-making score and the lowest mean participator decision-making score and were more likely to defer their final treatment decision to their physicians than English-speaking Latinas or whites (26%, 13%, 18%, p<0.05). - More than half of the women in the study reported that they had made their decisions together with their physicians (61%). - Spanish-speaking Latinas were the least likely to report making decisions together with their physicians (58% Spanish-speaking Latinas vs., 68% English-speaking Latinas and 60% whites, p=0.02) and the most likely to report that the physician had made most of the treatment decisions compared to English-speaking Latinas and whites (26%, 13%, and 18%, p=0.02). - Spanish- and English- speaking Latinas were less likely than whites to report making most of the treatment decisions alone (16%, 19%, and 22%, p=0.02). - Spanish-speaking Latinas were the least likely to report participatory decision-making compared to English-speaking Latinas and whites as measured by mean scores on the Participatory Decision-making scale (3.0 vs. 3.4 and 3.2, p=0.02). - Among Spanish-speaking Latinas, 69% reported having an interpreter present when discussing treatment options with their physicians. - Spanish-speaking Latinas reported lower satisfaction with treatment decision-making (OR 0.4; CI 95%, 0.2-0.8) and expressed more regret than whites (OR 6.2; CI 95%, 3.0-12.4). - More participatory decision-making increased the odds of satisfaction (OR 1.5; CI 95%, 1.3-1.8) and decreased the odds of treatment regret (OR 0.8; CI 95%, 0.7-1.0), independent of ethnicity-language. |
| Sanders [31]  2014  USA | Questionnaire | To explore psychological and psychosocial distress in patients with DCIS when compared to patients with early invasive breast cancer. | 32 patients were enrolled in the study.  Patients ranged in age from 22-73 years for DCIS and from 37-74 years for EIBC. | - Both groups were asked to rate their perception of breast cancer risks on a five-point Likert-type scale. Overall, responses showed little difference between the study groups. - Despite the different medical risks of recurrence, the means of DCIS and early invasive breast cancer groups showed low levels of psychological distress when asked to describe their perceived level of risk for the cancer returning to the same breast, spreading to the opposite breast, and spreading to other parts of the body. - Patients in this study indicated an optimistic outlook for the future regardless of the difference in diagnosis. - The mean scores for DCIS and early invasive breast cancer were similar when examining perceived risk of dying on a five-point Likert-type scale. The responses suggested that both sets of patients experienced low levels of distress in terms of their disease causing death. - Both groups received strong support and understanding from their spouses or significant others. When asked about their partner's understanding of the disease, both groups felt that their partner's understanding was moderate to well informed. Both groups felt that they received moderate to high support from their spouses or significant others. |
| Bloom [32]  2013  USA | Qualitative study  (interviews) | To compare quality of life domains between ethnic and language groups within a breast cancer context. | 396 Euro-American women and 349 Latina women; 156 were interviewed in English and 193 in Spanish, with a median of 2 years after diagnosis.  Median age for Euro-American, Latina English and Latina Spanish were 57.1, 56.7 and 54.9 years respectively. | - Younger age, no partner, and lower income were related to lower quality of life in various domains. - Physical comorbidities were associated with lower physical, psychological, and social quality of life; lingering effects of surgery and prior depression were associated with lower quality of life in all domains. - English-speaking and Spanish-speaking Latinas reported higher spiritual quality of life, and Spanish-speaking Latinas reported lower social quality of life than Euro-American women. |
| Lopez [33]  2013  USA | Qualitative study  (interviews) | To compare posttreatment care by ethnicity-language and physician specialty among Latina and White women with DCIS. | 745 (396 White, 349 Latinas) women diagnosed with DCIS between 2002 and 2005, aged ≥18 years, identified through the California Cancer Registry completed a telephone interview in 2006.  Mean age was 57 years and women over 60 comprised more than 1/3 of the sample. | - The majority of women were either married or living with a partner, were employed, had attended college or higher, and were privately insured. Spanish-speaking Latinas were less affluent, less educated, had lower rates of employment, and were less likely to be privately insured than English-speaking Latinas and Whites. - Of 742 women, most (90%) had at least one clinical breast exam. - Among women treated with breast-conserving surgery (n=503), 76% had received at least two mammograms. - While 92% of all women had follow-up rates (84%) of all groups. - Lifestyle counseling was low with only 53% discussing exercise, 43% weight, and 31% alcohol in relation to their DCIS. - In multivariable analysis, Spanish-speaking Latinas with breast-conserving surgery had lower odds of receiving the recommended mammography screening in the year following treatment compared to Whites (OR 0.5; 95% CI, 0.2-0.9). - Regardless of ethnicity-language, seeing both a specialist and primary care physician increased the odds of mammography screening and clinical breast exam exercise, weight, and alcohol use, compared to seeing a specialist only. |
| Bober [34]  2013  USA | Questionnaire | To characterize sexual functioning in women recently diagnosed with DCIS, as well as upon 9 and 18 month follow-up. | Of the 815 women with newly diagnosed DCIS identified from the Rapid Case Registry, 764 patients were deemed eligible for the study and 487 enrolled on the study (64% of eligible patients). Of these women, 304 (65%) reported being sexually active and completed the survey.  Median age=50.3; range=26.0-84.0. | - Regarding the Multidimensional Body-Self Relations Questionnaire-Appearance Scale, scores at baseline (mean=3.5, SD=0.8) as well as 9 month (mean=3.6, SD=0.8) and 18 month (mean=3.5, SD=0.8) follow-up were very stable. - Scores among women in the study were in line with reported population norms (mean= 3.38, SD=0.85) across all time points. - Scores for sexual arousal and sexual satisfaction were also similarly stable across all time points (sexual arousal: means=13.9014.0, SD=3.5-3.8; sexual satisfaction: means 12.1-12.3, SD=2.1-2.2) and were very consistent with findings in large-scale studies of breast cancer survivors as well as in healthy postmenopausal women. - There was no significant difference on sexual satisfaction between patients who were treated with tamoxifen and those who were not. - Of the 86 patients who had mastectomy, there were no differences in sexual satisfaction for patients who had reconstruction compared with patients who did not. - However, patients with mastectomy and no reconstruction reported significantly higher average sexual satisfaction than patients who did not have mastectomy (mean=1.25, SE=0.48). - Lower scores of sexual satisfaction were reported by women who had mastectomy with reconstruction at baseline. |
| Parikh [35]  2013  USA | Qualitative (interviews) | To evaluate knowledge of DCIS among a cohort of English and Spanish speaking Latina and English speaking non-Latina white women previously treated for DCIS. | Participants met the following inclusion criteria: (1) diagnosed with histologically confirmed DCIS in 2002-2005; (2) self-identified as Latina or non-Latina white; (3) English or Spanish-speaking; (4) 18 years of age or older; and (5) no subsequent diagnosis of invasive breast cancer. Study recruitment took place between Jan 2005 and Sept 2006. Within each region and county, all Latina women were sampled. Given the larger number of non-Latina white women, they were selected randomly and matched to Latina cases by age (within 5-year increments), diagnosis period (within 6-month intervals), and county of diagnosis.  Of the 1231 women eligible for the study, attempts to contact them resulted in 319 refusals, 167 non-respondents, and 745 completed surveys. Whites had a higher completion rate than Latinas (67% and 55% respectively). From this group of 745, 710 patients answered the knowledge survey questions and were included in the study.  The mean age of the sample overall was 57 (range 27-78). | - Of the 190 Spanish-speaking Latina participants, 64% reported having someone present to interpret for them while with their breast cancer physician. Of these, 50% had a family member or friend interpret for them. 44% had non-interpreter clinic staff (e.g., a nurse or clerk) interpret, and 3% had a professional interpreter. Four participants did not report who did the interpreting. - Overall, less than half (41%) of the women were aware that DCIS is not life-threatening and only a third (32%) knew that mortality risk is the same for mastectomy and lumpectomy plus radiation. - English-speaking whites were more likely to know that DCIS is not life-threatening. - Spanish-speaking Latinas were most likely to correctly assess that women with DCIS have a higher chance of developing breast cancer in the future and that the mortality from DCIS after mastectomy was similar to that after lumpectomy with radiation. - Compared with English-speaking whites, both English-speaking and Spanish-speaking Latinas had significantly lower odds of knowing that DCIS was not itself life-threatening (OR, 95% CI 0.6, 0.4-0.9 and 0.5, 0.3-0.9 respectively). In contrast, Spanish-speaking Latinas had more than twofold higher odds of knowing that DCIS increases risk of future breast cancer (OR, 95% CI 2.6, 1.6-4.4) while there was no difference in knowledge between English-speaking Latinas and English-speaking whites. - Surgical treatment type was not independently associated with knowledge. None of the potential confounders except for age were associated with any of the knowledge outcomes. Age as associated with decreased odds of knowing the risk of future breast cancer (OR, 98% CI 0.98, 0.96-1.00). |
| Ruddy [36]  2013  USA | Questionnaire | To evaluate risk perceptions among women who had been diagnosed with DCIS around 5 years prior. | 816 with newly diagnosed DCIS were originally identified for this study and the final sample size was 181.  The median age of respondents was 54 years (range, 34-76). | - 24% of the participants perceived their risk for DCIS spreading to other places in their body to be at least moderate, 32% perceived at least a moderate risk for developing DCIS again within 5 years, 43% perceived at least a moderate lifetime risk for developing DCIS again, 27% perceived at least a moderate risk for developing invasive breast cancer within 5 years, and 38% perceived at least a moderate lifetime risk for developing invasive breast cancer. - Compared with the surveys conducted at enrollment and after 18 months, the proportion of women who perceived their risk to be at least moderate decreased for all outcomes other than spread to other parts of the body. - The median reported quantitative risks were 5% for DCIS recurrence within 5 years, 5% for invasive cancer within 5 years, 10% for DCIS over a lifetime, 10% for invasive cancer over a lifetime, and 9% for spread of DCIS to other parts of the body. - The factor most strongly associated with perceived risk in long-term follow-up was risk perception at 18 months. - Perception of developing invasive breast cancer within a lifetime was associated with socioeconomic factors. Women who were financially comfortable were less likely to perceive moderate or greater risk, as were women who were at least college graduates. Perceptions of DCIS spreading throughout the body were also associated with financial status: women who were financially comfortable were less likely to have a moderate or greater perceived risk for this event. - The proportion of participants who met the criteria for anxiety based on the Hospital Anxiety and Depression Scale score (≥11) was low and declined only slightly over time. Depression, defined as a Hospital Anxiety and Depression Scale score of ≥11, was only identified in one of the 181 eligible respondents. There was no association between any of the measures of risk perception and anxiety as measured using the Hospital Anxiety and Depression Scale. - The proportion of participants with an Impact of Event Scale score of ≥26, reflecting intrusive and avoidant thoughts about DCIS, decreasing substantially over time, from 23.8% at enrollment to 3.9% at 5 years. - The average Physical Health Component Summary score was 47.6 (SD, 6.7; median, 48.7; range, 15.7-57.8). The average Mental Health Component Summary score was 46.2 (SD, 5.8; median, 47.2; range, 23.6-60.3). Compared with the average score of 50 in the general population, these differences approximate a minimum clinically significant difference of 3 points of these measures. - Only 22% of evaluable women had a Physical Health Component Summary score <50 in long-term follow-up, whereas 54% had a Physical Health Component Summary score <50 at baseline. Controlling for other factors, women who felt that they had at least a moderate risk for DCIS spreading had a Physical Health Component Summary score that was 3 points lower than those who perceived their risk to be lower (95% CI, 0.9-5.3; p=0.005). |
| Sue [37]  2013  USA | Single Cohort | To determine rates of mastectomy and post mastectomy reconstruction among patients presenting with DCIS and to determine factors associated with these decisions. | A retrospective cohort study was performed on 196 consecutive female patients presenting with DCIS and receiving surgical treatment over this period at the Yale Breast Center.  The median age of all patients at the time of diagnosis of DCIS was 55.5 years (range, 35.8 to 88.9 years). | - Of the 199 patients who underwent surgery for DCIS, 48 (23.6%) were treated with mastectomy, and 152 (76.4%) underwent breast-conserving surgery. - Of the patients who underwent mastectomy, 14 (29.8%) opted for contralateral prophylactic mastectomy at the time of surgery. - Patients who opted for mastectomy were younger than those treated with breast-conserving surgery (median age, 51.3 vs 57.4 years; P=0.013). Additionally, patients undergoing mastectomy were more likely to have higher grade tumors compared with patients undergoing breast-conserving surgery (50.0% vs 34.1% grade 3, P=0.008). - Among patients treated with mastectomy, those who opted for reconstruction were younger than those who did not receive reconstruction (49.4 vs 56.9 years, P=0.024). |
| Livaudais [38]  2012  USA | Qualitative (interviews) | To explore a variety of factors associated with discussion, use, and discontinuation of adjuvant hormonal therapy for DCIS, including patient, tumor, and treatment-related characteristics and physician-patient communication factors. | 744 women diagnosed with DCIS from 2002 through 2005, aged ≥18 years, of Latina or non-Latina white race/ethnicity.  Mean age was 56.5 years. | - Although 83% of women discussed adjuvant hormonal therapy with a physician, 47% used adjuvant hormonal therapy, and 23% of users reported discontinuation by a median of 11 months. - The most frequently reported reasons for discontinuation were the experience of side effects (69%) and recommendation by a physician to stop using the therapy for medical reasons (45%). - In multivariable adjusted analyses, Latina Spanish speakers were less likely that white women to discuss therapy (OR 0.36, 95% CI 0.18-0.69) and more likely to discontinue therapy (OR 2.67, 95% CI 1.05-6.81). - Seeing an oncologist for follow-up care was associated with discussion (OR 5.10, 95% CI 3.14-8.28) and use of therapy (OR 4.20, 95% CI 2.05-8.61). - Similarly, physician recommendation that treatment was necessary vs. optional was positively associated with use (OR 11.2. 95% CI 6.50-19.4) and inversely associated with discontinuation (OR 0.38, 95% CI 0.19-0.73). |
| Kennedy [39]  2012  UK | Qualitative (interviews) | To describe the evolution of women's perceptions and experiences of DCIS from the period near to diagnosis to 1 year later. | 45 women participated in the initial interviews with a mean age of 58.8 years and 34 of them were in a relationship.  27 also participated in follow-up interviews. | - Participants' main response to their diagnosis was a feeling of relief, especially given DCIS' early, contained state and positive prognosis. - Initial perceptions of DCIS were informed by the terminology used by health professionals. Often these were 'pre-cancer(ous)' or 'DCIS', but other explanations included 'early stages of breast cancer', which contradicted with other being told 'it wasn't cancer'. - This confusion was enhanced by conflicting information between what they were verbally told and the written information they were given, which was often primarily designed for women with invasive breast cancer. - One of the most enduring emotions was the feeling of ongoing risk, which ranged from fleeting concerns to significant intrusive thoughts. - Persistent concerns were voiced about the possibility of recurrence or development of invasive breast cancer and some felt they were living in a cloud of vulnerability and uncertainty about the future. - This concern continued in the follow-up interviews and led women to constantly seek reassurance. - Overall, DCIS had a profound impact on some women, but this was juxtaposed with relief that their disease was caught early. - Women's own lack of awareness and confusion about DCIS made telling others challenging. - Some women used the terminology used by their clinicians, but several were concerned that this would lead others to perceive the diagnosis as serious and assume it was breast cancer. - Some specifically tried to reduce their family's concern by using the term 'pre-cancer(ous)' to minimize its significance. - One year post diagnosis, most participants felt they no longer needed health professionals' support, but were grateful if it was still available. - Breast-cancer nurses were highlighted as providing invaluable support in most cases. Most women tried to put the experience behind them. - There were mixed feelings about the value of DCIS-specific peer support. Some felt it would have helped them to make sense of DCIS, but others were concerned about meeting women with different views of the condition. |
| Jeffe [40]  2012  USA | Mixed method (survey and interviews) | To examine the impact of a DCIS diagnosis on quality of life outcomes, by comparing women with DCIS, women with early-stage invasive breast cancer, and a comparison group of age-matched women without a history of breast cancer. | 549 patients (71.1% of 772 invited) and 547 controls (57.8% of 946 invited) were enrolled. Retention was high with 1011 participants completing T4 (92.2% overall; 514 (93.6%) patients, 497 (90.9%) controls).  184 patients had DCIS and 365 patients had early-stage invasive breast cancer.  The mean age of DCIS patients was 57.2 years. The mean age of early-stage invasive breast cancer patients was 58.9 years. | - Controls reported better quality of life at T1 (6.7 weeks) on each subscale compared with DCIS patients (each P<0.02) and with early-stage invasive breast cancer patients (each P<0.0001). - Subscales: physical functioning, role limitations due to physical problems, role limitations due to emotional problems, energy/fatigue, emotional well-being, social functioning, general health. - At T1 (4-6 weeks after treatment), DCIS patients reported better quality of life than early-stage invasive breast cancer patients on the role limitations due to physical problems (P=0.0021), energy/fatigue (P=0.0231), and social functioning (P=0.0006) subscales. Both DCIS and early-stage invasive breast cancer patients showed improvements in quality of life over the 2 year follow-up. - By T2 (6 months after treatment), there were still significant differences by diagnostic group overall in physical functioning (P<0.0001), role limitations due to physical (P<0.0001) and emotional (P<0.0053) problems, energy/fatigue (P=0.0005), social functioning (P<0.0001), pain (P=0.0087), and general health (P<0.0001). Only emotional well-being did not differ significantly by diagnostic group at T2. All 8 subscales at T2 differed significantly between early-stage invasive breast cancer patients and controls (each P<0.005). In addition, DCIS (P=0.0118) and early-stage invasive breast cancer (P<0.0001) patients each reported worse physical functioning compared with controls at T2, and DCIS patients reported significantly better quality of life on role limitations due to physical problems (P<0.0001), energy/fatigue (P=0.0008), social functioning (P=0.0004), pain (P=0.0206), and general health (P=0.0392) than early-stage invasive breast cancer patients at T2. - By T3 (1 year after treatment), there were still significant differences by diagnostic group overall in physical functioning (P=0.0337), role limitations due to physical problems (P=0.0379), energy/fatigue (P=0.0010), and general health (P=0.0011). DCIS patients no longer differed significantly from controls on any of the eight quality of life subscales, but early-stage invasive breast cancer patients still reported worse QOL than controls on physical functioning (P=0.0095), role limitations due to physical problems (P=0.0155), energy/fatigue (P=0.0131), and general health (P=0.0002). Moreover, early-stage invasive breast cancer patients reported worse energy/fatigue compared with DCIS patients at T3 (P=0.0004). - By T4 (2 years after treatment), the main effect of diagnostic group was significant only for physical functioning (P=0.0005) and general health (P=0.0059). Early-stage invasive breast cancer patients still reported worse physical functioning (P=0.0001) and general health (P=0.0017) than controls. |
| Davey [41]  2011  Australia | Mixed methods | To gain insight into the characterisation of DCIS from the perspective of consumers and surgeons. | 231 women diagnosed with DCIS in 2006/2007 in Victoria, Australia.  63 treating surgeons completed a mailed survey | - The main outcome measures were: women's diagnostic experience, women's and surgeons' description of DCIS, women's understanding of DCIS, confusion and worry about the disease and risk perceptions. - While the majority of women had not heard of DCIS prior to diagnosis, most reported a positive diagnostic experience. - Surgeons' and women's description of DCIS were consistent. - Women understood that DCIS is a contained disease (86%), can progress (88%) and treatment aims to prevent invasive cancer (97%). - However, only 13% understood that DCIS alone cannot spread to other parts of the body. - A quarter of the women were confused about the risk of DCIS spreading. - Younger women had more concerns about developing breast cancer (p=0.008) and the disease spreading (p=0.002) and rated their risk of invasive disease higher (p=0.007). - Most women diagnosed with DCIS in 2006/2007 understand the 'early, contained nature' of the disease, but understanding of the 'non-invasive' nature of DCIS could be improved. |
| De Morgan [42]  2011  Australia | Questionnaire | To assess knowledge, satisfaction with information, decisional conflict and psychological morbidity amongst women diagnosed with DCIS and to explore the factors associated with less knowledge and greater confusion about DCIS. | 144 women diagnosed with DCIS were sent a survey.  The mean age of participants was 56 years old.  Most participants lived in a city, were currently in a relationship, and spoke English as their first language.  Approximately half of the participants had a tertiary education and were currently employed. | - This study found misunderstanding and confusion amongst women diagnosed with DCIS and a desire for more information about their breast disease. - Approximately half of participants worried about their breast disease metastasizing; approximately half expressed high decisional conflict; 12% were anxious and 2% were depressed. - Logistic regression analysis demonstrated that worry about dying from the breast disease was significantly associated with not knowing that DCIS could not metastasize (OR 3.9; 95% CI 1.03-14.25); and confusion about whether DCIS could metastasize was significantly associated with dissatisfaction with information (OR 12.5; 95% CI 3.8-40.2). |
| De Morgan [43]  2011  Australia | Mixed methods | To assess patient and clinician perceptions of a developed DCIS communication aid. | 18 Australian women with DCIS with an average age of 63 years, participated in structured interviews and 7 clinicians (5 breast surgeons and 2 radiation oncologists) completed surveys. | - All (100%) or most (94%) women felt the communication aid would help women to understand their diagnosis and treatment and would assist in communication between doctor and patient without increasing anxiety. - Most women liked the content and format of the communication aid, including the diagrams. - Most women liked that DCIS was described as "not breast cancer as we commonly understand it". - All (100%) or most (86%) clinicians felt the communication aid would help women to understand their diagnosis and treatment and assist them to communicate with women newly diagnosed with DCIS without increasing anxiety. - All of the clinicians said that they liked the content of the communication aid. - Most clinicians used all of the information and diagrams in the communication aid in their order of publication. - Most clinicians (86%) reported that they would use the communication aid regularly and most clinicians reported that they thought women with DCIS would like the communication aids. - Most clinician’s reported that the communication aid would not make their consultations too long or change their consultation style. - Most clinicians approved of DCIS being described as "not breast cancer as we commonly think of breast cancer". |
| Kaplan [44]  2011  USA | Qualitative (interviews) | To identify the role of ethnicity and language in the receipt of reconstruction, the relationship between system-level factors and the receipt of reconstruction, and women's reasons for not undergoing reconstruction. | Women aged 18 and older, who self-identified as either Latina or non-Latino white, were diagnosed with DCIS between 2002 and 2005, and resided in one of right California Cancer Registry regions.  The survey was successfully completed by 745 women, 239 of whom had a mastectomy and represent the sample included in this study. | - Mean age was 54 years. - The majority of respondents (71%) were married or living with a partner. 41% reported having a close relative with breast cancer, and 25% indicated having a major co-morbidity. - Excellent or very good health was reported by 41% of the sample. - A greater proportion of whites and English-speaking Latinas indicated excellent or very good health as compared to Spanish-speaking Latinas. - The majority of whites and English-speaking Latinas were privately insured, while this was not the case for Spanish-speaking Latinas. - Whites had a higher completion rated than Latinas (67 and 55%, respectively). Analysis included descriptive statistics and logistic regression modeling. - A greater proportion of whites had reconstruction (72%) compared to English-speaking Latinas (69%) and Spanish-speaking Latinas (40%). - Multivariate analysis showed that women who were aged 65 and older, unemployed, and had a lower ratio of plastic surgeons in their country were less likely to have reconstructive surgery after mastectomy. - The most frequent reasons mentioned not to receive reconstruction included lack of importance (85% whites, 80% English-speaking Latinas, and 70% Spanish-speaking Latinas) and desire to avoid additional surgery (74% whites, 67% English-speaking Latinas, and 65% Spanish-speaking Latinas). |
| Liu [45]  2011  USA | Mixed method (surveys and interviews) | To identify psychosocial, demographic, disease-related, and treatment-related correlates for fear of cancer recurrence in a cohort of women diagnosed with DCIS and early invasive breast cancer. | 772 patients met the inclusion criteria and were identified prospectively using the medical record and surgical pathology reports. 587 patients consented to participate, and 549 (71%) were eligible. Of the 549 participants, 514 (94%) completed the 2 years follow-up interview. Overall, 506 women were analyzed.  The average age was 58 years. | - Univariate analysis showed that patients who were diagnosed with stage IIA breast cancer, had completed chemotherapy, or had elevated anxiety or elevated depressive symptoms at baseline reported greater fear of cancer recurrence 2 years after definitive surgery (each P<0.01). - Higher fear of cancer recurrence scores were moderately correlated with younger age, more severe surgical side effects, and lower social support reported at baseline. - The average fear of cancer recurrence score was low in the sample, with a mean of 2.0 on a 1-6 scale. However, 24.8% (123/506) of patients reported moderate levels, and 4% (22/506) reported high levels of fear of cancer recurrence. Moderate-to-high fear of cancer recurrence (range 3.0-6.0) was reported by 29.0% of DCIS patients, which was not significantly different from fear of cancer recurrence reported by stage IIA patients (38.7%, {=0.13) or stage II patients (26.0%, P=0.50). - More highly educated patients reported less fear of cancer recurrence and patients who had elevated depressive symptoms at baseline reported higher fear of cancer recurrence. - The effects of education and elevated depressive symptoms on fear of cancer recurrence were not observed at the 6 month and 2 year follow-ups. - Breast-conserving surgery and more severe surgical side effects at 6 month and 2 year follow-ups were significantly correlated with higher fear of cancer recurrence, but were not correlated with fear of cancer recurrence at baseline. - Compared with patients who accurately perceived their risk of recurrence, patients who overestimated their risk of recurrence at the 2 year follow-up reported greater fear of cancer recurrence, and patients who underestimated their risk of recurrence at the 2 year follow-up reported less fear of cancer recurrence. |
| Prinjha [46]  2011  UK | Qualitative (interviews) | To explore how women who have a mastectomy for screen-detected DCIS make sense of their diagnosis and treatment options. | 35 women were recruited using a range of recruitment strategies and sources aiming for a diverse maximum variation sample of screen-detected DCIS, including various social-class and ethnic backgrounds. The study aimed to include people whose experience might be considered 'typical' as well as those with more unusual experiences.  20/35 women with screen-detected DCIS had a mastectomy; the rest had a wide local excision.  Ages ranged from 49 to 66 years. | - Four key themes were identified through interviews: 1. Understanding of routine breast screening. 2. Uncertainty about DCIS and its natural progression. 3. Uncertainty about whether a mastectomy is justified for DCIS. 4. Information gaps and treatment decisions. - 1. Women were concerned about the information they received before routine breast screening. None had heard of DCIS before their own diagnosis or were aware that early, symptomless breast cancers existed. A few of the women who had a mastectomy questioned the value of routine mammography if it could lead to the diagnosis of a condition that might not progress. - 2. Women often found DCIS a confusing diagnosis because of the different terms used to describe it and the uncertainty about its natural progression. Making decisions about treatment was particularly difficult because clinicians could not predict the likelihood of their DCIS becoming invasive disease. - 3. Women were often very shocked and upset to hear they would need a mastectomy and found this hard to square with their lack of symptoms. A few women felt that having a mastectomy would give them reassurance that all the DCIS had been removed and about recurrence. Others were reluctant to have a mastectomy for a condition that might never progress. With hindsight, many women said they would have liked more information about DCIS and the treatment options. Some women also said they would have liked specific information about why DCIS patients are given different treatments, why doctors have different approaches to treating the same condition, and the need for a mastectomy for DCIS. Women sometimes wondered why DCIS had to be treated so soon after diagnosis and suspected that their treatment may have been driven by a need to meet government targets. A few women felt that a mastectomy for DCIS was over-treatment and questioned why women with multifocal DCIS could not be monitored instead. - 4. Women who questioned the need for a mastectomy found that the answers and information they needed were often not known, making treatment decisions extremely difficult. In the absence of sufficient factual information, one woman said the decision she finally made was hugely influenced by talking with another woman who had had DCIS in the past. In order to make an informed choice about having a mastectomy for DCIS, women wanted specific information about the condition and a clear explanation of why a mastectomy was required. |
| Kennedy [47]  2010  UK | Questionnaire | To address the gaps in the existing literature by exploring the psychosocial impact of DCIS amongst UK patients during the first year following diagnosis. | The 43 women ranged between 34 and 87 years old (mean 60.2). | - Overall, the incidence of anxiety reduced over time, but 2 women consistently remained at case or borderline level, 2 worsened and 5 improved at 6 months but worsened at 9 months. - Depression scores showed a similar pattern; one woman consistently reported a 'case' level of depressive symptoms, one worsened and one improved at 6 months but then reverted to 'case' level. - 8 women (18.6%) reported no body image concerns at any point during the study. - There was a reduction in anxiety and depression from baseline to 6 months, which is maintained at 9 months. - There was no difference between the surgical groups in anxiety or quality of life. However, the surgical groups differed significantly in terms of body image distress: p=0.002. - Women who underwent an immediate reconstruction reported significantly greater overall body image distress than wide local excision patients (p=0.001) and marginally (p=0.055) higher levels than those who underwent mastectomy without reconstruction. The percentage reporting 'priority' levels of body image distress was also greatest amongst the immediate reconstruction group. |
| Lauzier [48]  2010  Canada | Questionnaire | To compare women diagnosed with DCIS to women with invasive breast cancer who had had or did not have chemotherapy, separately, in terms of psychological distress and health-related quality of life during the first year after treatment initiation. | 1397 women with breast cancer were identified. Of 962 patients meeting a previous study's eligibility criteria, 829 (86.2%) consented to participate and completed the 1 month interview and 800 of the 962 initially eligible women (83.2%) completed all three interviews (1, 6, and 12 months after treatment).  13.4% (n=107) had DCIS and 86.6% (n=693) had invasive breast cancer. | - Compared to women with invasive disease, whether or not they had had adjuvant chemotherapy, women with DCIS reported slightly lower levels of distress at each time point, but the mean differences between groups were small, not statistically significant, and effect sizes were mostly negligible or very small (ES from -0.03 to -0.21). - Psychological distress declined in all three groups but the pattern of decline differed according to the type of breast cancer (P for the time-type of disease interaction: 0.028). - For the mental component scale, differences between women with DCIS and those with invasive breast cancer were even smaller and all effect sizes negligible (ES from -0.1 to 0.15). - Finally, women diagnosed with DCIS reported significantly higher levels of physical health on the physical component scale, compared to women with invasive cancer. - Sizable differences were seen primarily for comparisons of DCIS to invasive disease plus chemotherapy (ES=0.82; ES=0.70; ES=0.41 at 1, 6, and 12 months following treatment initiation, respectively), but only at the first interview when DCIS was compared to invasive disease without chemotherapy (ES=0.55; ES=0.17; ES=0.10 at 1, 6, and 12 months following treatment initiation, respectively; P for the time-type of disease interaction <0.0001). |
| Liu [49]  2010  USA | Qualitative (interviews) | To assess the accuracy of early-stage breast cancer patients' recurrence risk perceptions and identify the demographic, clinical, and psychosocial characteristics associated with the accuracy of their perceived risk. | Of 772 eligible patients, 549 (71%) completed the baseline interview 4-6 weeks after surgery; of these 549, 537 (98%) completed the 6 month post-surgery interview, and 527 (96%) completed the 12 month post-surgery interview. The analysis included 531 patients whose perceived risk could be assessed at either or both the 6 and 12 month follow-up.  179 patients had DCIS and 352 had early invasive breast cancer. | - At the 6 month interview, 17% accurately perceived their risk, 44% underestimated their recurrence risk, and 21% overestimated their risk. - This pattern of recurrence risk perceptions changed 6 months later (P=0.0143); 66% of 87 patients with accurate risk perceptions at the 6 month interview inaccurately perceived their risk and only 17% of patients with inaccurate risk perceptions at the 6 month interview accurately reported their risk at 12 months. - Patients with a diagnosis of DCIS, lower social support, and greater anxiety were more likely to overestimate their risk. (Early invasive breast cancer: OR=1.00; DCIS: OR=1.76; 95% CI=1.11-2.79). - Patients who were non-white and who underwent radiotherapy were more likely to underestimate their risk. - Knowledge of type of breast cancer was not significantly associated with overestimating or underestimating one's risk. - Older patients and patients who did not know their type of breast cancer were more likely to be uncertain about their risk. |
| Liu [50]  2010  USA | Qualitative (interviews) | To characterize the trend in patients' perceived risk of recurrence and its relation to a variety of demographic, clinical, and psychosocial factors. | 549/772 eligible patients (71%) completed the first interview. Of the 549 participants, 537 (98%) completed the 6 month follow-up, 528 (96%) completed the one year follow-up, and 514 (94%) completed the 2 year follow-up.  At baseline, 182 (34%) of participants had DCIS. | - The percentage of patients who reported being uncertain about their risk of recurrence varied at each interview, ranging from a high of 16.4% at the 6 month interview to a low of 8.8% at the 2 year follow-up. - At the first interview, 16% of patients reported having no lifetime risk of recurrence (0%) and another 16% reported their risk to be >50%. - Notably, 15% of DCIS patients reported their risk to be >50%. - Perceived risk did not differ significantly by cancer stage at the first interview. - For the effect of stage at any given time, the marginal estimated probability of reporting each perceived risk category did not differ significantly between DCIS and stage I patients (P=0.6381). |
| Perez [51]  2010  USA | Interviews | To assess the psychometric properties of a new measure of sexual problems and to identify differences in sexual problems by diagnostic group (DCIS, stage I, stage IIA, and controls). | 549 (71.1% of 772) patients and 547 (57.8% of 946) controls were enrolled and completed the first telephone interview. Of the 1096 participants who completed the first interview, 25 patients and 38 controls were excluded from this analysis because they responded to less than 50% of the items in the interviews.  524 patients and 509 controls were included in the analysis. Of the 1033 participants who completed one or more telephone interviews, 1005 completed the second interview, 989 completed the third interview, and 959 completed the fourth and final interview. | - The numbers of women who had sexual-problem data available for analysis varied at each interview for the three sexual-problem measures at follows: 880 to 898 women at T1 (4-6 weeks after treatment), 833 to 859 women at T2 (6 months), 812 to 854 women at T3 (1 year), and 793 to 820 women at T4 (2 years). - Controls were more likely to report sexual problems on the 9-item measure (T2-T4), on the problems with sexual attractiveness subscale (T4), and on the problems with sexual interest/enjoyment subscale (T3-T4) compared with T1. - Patients with stage I breast cancer were less likely to report problems with sexual attractiveness at T2 to T4 compared with T1. - Patients with DCIS and with stage IIA breast cancer with neither more nor less likely to report changes over time in problems with any of the three sexual-problem outcomes compared with their measures at T1. - In addition, patients with DCIS and stage IIA breast cancer did not differ significantly from controls at T1 in terms of any of the sexual-problem outcomes; however, patients with stage I breast cancer were 2.7 time more likely at T1 to report problems with sexual attractiveness compared with controls. - Patients who had a mastectomy were more likely to report sexual problems at T4 compared to T1, but no other significant interaction effects between surgical procedure and time were observed. - There also were no significant main effects of type of surgical procedure at T1. |
| Piot-Ziegler [52]  2010  Switzerland | Questionnaire | To understand the consequences of body deconstruction through mastectomy on corporality and identity in women with breast cancer. | Nineteen women aged 37-62 diagnosed with breast cancer participated in this study.  Carole, Elisa, Delphine had DCIS. | *Illness and mastectomy: A challenge to body integrity and corporality*   - All 19 women describe cancer, mastectomy and other treatments as a challenge to body integrity and all three DCIS patients describe it as stigmatizing. Elisa and Delphine described it as an affront to femininity. - All women talked about the sudden intrusion of illness and about its medical and physical consequences. Delphine was not much concerned about her appearance before cancer was diagnosed. She mentioned that she was less concerned about mastectomy because she was older. Also, she considered her body as something of lesser importance. Before mastectomy, Elisa mentioned the necessity of mourning their breasts, and that this process lies within a time perspective. - Carole and Elisa expressed concerns about future emotional or psychological negative reactions, when it would be necessary to face a modified physical appearance after surgery. - Delphine seemed at first to be less disturbed by the physical and psychological consequences of the surgical intervention, but in the course of the interview she talked about her distress when confronting a modified body, on the visual but also on the sensual level. For Elisa and Delphine, mastectomy represented a mutilation and amputation. - All three women spoke about their fears and about the feeling of emptiness associated mastectomy. Carole described their concerns about losing the areola, which they consider as being central to the breast. - Carole and Elisa described the asymmetry related to mastectomy, where physical loss of balance and psychological crisis meet. Elisa and Delphine talked about the loss of breasts as something that belittles them.   *Body deconstruction: A challenge to the woman’s identity*   - Elisa and Delphine feared that they would no longer be or feel like a woman. Elisa expressed the feeling that mastectomy would prevent her from becoming a mother. Delphine talked about her illness as a threat not only for her but also for her daughter. Elisa and Delphine mastectomy induced a discrepancy between what is socially expected from a woman's appearance and their own reality.   *Body deconstruction and relationships to others*   - Elisa and Delphine feared that people would notice the missing part of their body even when they were dressed, or that people might react in a negative way to their modified physical appearance. Elisa and Delphine, in order to avoid shocking or hurting other people, felt the need to restrain their personal clothing choices or freedom of movement, and conceal their bodies from visual exposure.   *Body reconstruction: An identity challenge*   - The possibility of reconstruction was of some comfort for Carole and Delphine. - Humour helped Delphine face uncertainty about the result of reconstruction, and to withstand anxiety and physical and psychological suffering. - Carole reported that cancer had led her to re-evaluate her existential priorities. It modified her way of interacting with other people, leading her to be closer and more affectionate. |
| Sackey [53]  2010  Sweden | Questionnaire | To investigate and compare long-term health-related quality of life, body image, and emotional reactions in women with DCIS treated with different surgical methods. | All women who took part in the Swedish National DCIS study during the years 1991-1999 in the County of Stockholm were eligible. 162 women were included in the study and 131 (81%) responded to questionnaires.  The median age was 58.5 years (range 40-77) for women treated with mastectomy and immediate breast reconstruction, 65 years (range 55-83) for those treated with sector resection alone, and 64 years (range 48-89) for women treated with sector resection and postoperative radiotherapy. | - 47 women had mastectomy and immediate breast reconstruction, 51had sector resection alone, and 64 had section resection and postoperative radiotherapy. - The score for mental health was statistically significantly higher in the sector resection alone group than in the other two groups. - Women in the mastectomy and immediate breast reconstruction group scored statistically significantly higher on physical functioning and bodily pain than their age-adjusted norm group. - Overall, statistically significant differences between the three study groups were found for six of the items (self-conscious, less physically attractive, less feminine, less sexually attractive, dissatisfied with body, dissatisfied with scars) with larger proportions of women in the mastectomy and immediate breast reconstruction group reporting problems. - Overall, women in all three study groups appeared to have a very satisfactory quality of life in the long term, similar to women in the general population. |
| Schroen [54]  2010  USA | Questionnaire | To identify the relative importance of different information sources on surgeons' decision-making when faced with scientific uncertainty. | Of 2188 survey recipients (American College of Surgeons members), 923 responded, with 460 responses meeting eligibility criteria. | - Surgeons rated 7 sources of information for each area of clinical uncertainty. - In each area of uncertainty, the "expert opinion of someone you regard as a leader in the field" ranked as the most influential source of information, and published guidelines or consensus statements were ranked as the second most influential source of information. - Academic surgeons, however, consistently ranked published data as more influential than expert opinion. - When compared among various surgeons and practice characteristics, the area of uncertainty that generated the most consistent ratings was the use of sentinel lymph node biopsy in DCIS. In this area, only academic surgeons did not rate expert opinions as most influential in decision-making; academic surgeons rated observational studies highest. - The area of uncertainty generating the most diverse ratings was use of post-lumpectomy radiation therapy for DCIS. - Randomized controlled trial data received the highest average ratings among women, academic surgeons, surgeons with oncology training, surgeons with professional society memberships, and surgeons with new breast cancer patient volumes of >50 per year. Men, surgeons in practice fewer than 20 years, and surgeons without oncology training rated guidelines or consensus statements as most influential in this area. |
| Tuttle [55]  2009  USA | Single cohort | To evaluate the contralateral prophylactic mastectomy rates for women with DCIS and identify factors significantly associated with contralateral prophylactic mastectomy use. | 51,030 women with unilateral DCIS who were treated with surgery from 1998 to 2005 were identified.  Ages ranged from 18 to 79 years. | - Young patient age was associated with significantly higher contralateral prophylactic mastectomy rates on logistic regression analysis. For patients younger than 40 years, the contralateral prophylactic mastectomy rate was 12.6% for all surgically treated patients and 25.9% for patients undergoing mastectomy. - White race, recent year of diagnosis, and the presence of lobular carcinoma in situ were also significantly associated with higher contralateral prophylactic mastectomy rates among all surgically treated patients and all patients undergoing mastectomy. - Patients with larger tumors and higher grade were more likely to undergo bilateral mastectomy; however, among patients undergoing mastectomy, those with smaller tumors and lower grade were more likely to choose contralateral prophylactic mastectomy. - During the study period, the rate of breast-conserving surgery increased (1998, 66.9%; 2005, 71.5%), whereas the rate of unilateral mastectomy decreased (1998, 30.9%; 2005, 23.3%). The contralateral prophylactic mastectomy rates significantly increased during the study period. - Among all surgically treated patients (including breast-conserving surgery), the contralateral prophylactic mastectomy rate increased by 148% from 1998 (2.1%) to 2005 (5.2%). Among patients undergoing mastectomy (excluding breast-conserving surgery), the contralateral prophylactic mastectomy rate increased by 188% from 1998 (6.4%) to 2005 (18.4%). |
| Kennedy [56]  2009  UK | Questionnaire | To investigate the terminology, perception and experiences of DCIS among UK health professionals, and to compare responses across a variety of professional groups. | 296 health care professionals involved with the treatment of DCIS.  90 surgeons, 51 breast-care nurses, 47 pathologists, and 40 radiologists. | - Excluding pathologists, almost one-third described low grade DCIS as a type of 'cancer', and an additional 20% described it as 'cancerous', 'malignant' 'cells' or 'changes'. - In contrast, 21.8% explained it as a 'pre-cancer(ous) condition' or 'pre-cancer(ous) cells' and 17.2% as an 'abnormality', 'abnormal cells' or 'cell changes', in which there was no mention of cancer or malignancy. - Oncologists were significantly less likely to use the terms that mentioned cancer or malignancy (p=0.01) and compared to other professions, were significantly less likely to view DCIS as breast cancer (p=0.024). Of the 246 responses (excluding pathologists who did not have direct patient contact), 54 professionals (22%) found it somewhat or very difficult to explain DCIS to patients, whereas 46.7% indicated it was not difficult, and the remaining 31.3% were undecided. - However, in contrast and surprisingly given the previous agreement of low-medium perceived risk, 51.4% found DCIS more difficult to explain to patients than invasive breast cancer (only 9% found DCIS easier to explain). - The challenge of DCIS for patients that was most frequently reported by professionals was 'understanding the condition'. |
| Kennedy [57]  2008  UK | Qualitative (interviews) | To explore women's experiences of DCIS. | 16 women previously diagnosed and treated for DCIS. | - Reactions to the diagnosis primarily entailed shock and distress. This was shared by all the women, but appeared especially prominent in asymptomatic patients. - Most women initially express indifference when they were recalled for further tests or at the first sign of a problem. Therefore most were ill-prepared, the diagnosis was unexpected and the shock was enhanced by the invisibility of DCIS and because they felt fit and healthy, not ill. - The majority of women had never heard of the condition prior to their diagnosis - only one participant had been aware of it. - Uncertainty resonates throughout the women's accounts of the condition itself and the information available. - Women also recognised the uncertainty that their diagnosis was 'only' DCIS, since there was still a possibility of invasive cancer being identified. Unfortunately the information provided to them did not alleviate this uncertainty. - Although most women reported receiving basic information about DCIS, this was often limited to the first consultation or one leaflet amongst others that focused primarily on invasive breast cancer. - Participants reported considerable variation in how health professionals viewed and described the condition, but one of the strongest feelings that emerged was that, at times, the medical staff treated their diagnosis as inferior. - Acceptance of treatment appeared to be strongly linked to the women's perception of DCIS and the uncertainty surrounding the condition. - Four participants delayed their treatment while they researched the condition. - Women talked vividly about the initial period of shock being a surreal time and some experienced feelings of denial at this stage. |
| Partridge [58]  2008  USA | Questionnaire | To identify a cohort of women newly diagnosed with DCIS and to evaluate risk perceptions and psychosocial outcomes at study enrollment and over the course of the ensuing 18 months. | Consecutive women with newly diagnosed (≤6 months) DCIS were identified from systematic review of pathology reports at participating regional hospitals in Eastern Massachusetts. After a woman with newly diagnosed DCIS was identified, her physician was contacted to obtain permission to invite her to participate in the study. Eligible women were then invited by mail to participate in the study. Women were eligible if they had a diagnosis of DCIS and had either not yet completed surgical treatment or had undergone surgical treatment within 3 months before enrollment.  Of the 764 eligible patients, 487 (64%) enrolled and completed a questionnaire. 426 (87%) completed it at 9 months, and 392 (80%) at 18 months.  The mean age of enrolled women was 53.9 years. | - When women were asked to select the type of physician who was most influential in their care, 83% selected a surgeon, 10% a medical oncologist, 5% a radiation oncologist, and 2% another subspecialist. - 97% of respondents indicated that their perception of communication with their most influential physician was good, very good, or excellent. - 88% of patients indicated that they were satisfied with the care they received for their DCIS. - 77% of participants reported having seen a medical oncologist during their care. - Women reported a high degree of social support at enrollment, with a median Medical Outcomes Study Social Support Scale Score of 92.1 (range=13.2-100.0). - At enrollment, 10% of women reported substantial anxiety and 2% reported depression by the Hospitalized Anxiety and Depression Scale. McNemar's test comparing the proportion of patients with anxiety at enrollment at those at 18 months suggested that fewer women were anxious over time (P=0.06). The difference in mean Hospitalized Anxiety and Depression Scale anxiety scores between baseline and 18 months was highly statistically significant (-0.46, 95%CI=-0.79 to -0.14; P=0.006). - When women were asked to consider their DCIS when answering Revised Impact of Event Scale questions, the mean overall Revised Impact of Event Scale score was 15.9 at enrollment, indicative of a substantial degree of DCIS related intrusive or avoidant thoughts. Revised Impact of Event Scale scores decreased over time: the estimated mean decrease in Revised Impact of Event Scale between enrollment and 18 months using a mixed model revealed a statistically significant improvement (mean=-5.9, 95% CI=-7.1 to -4.7; P<0.001). - Women were queried about their perceived future breast cancer risks both at enrollment and at follow-up. At enrollment, 54% of women believed that they had at least a moderate likelihood of developing DCIS again in the next 5 years, and 68% believed that they had at least a moderate likelihood of developing DCIS again in their lifetime. 39% of women believed that there was at least a moderate likelihood of developing invasive breast cancer in the next 5 years, and 53% believed that there was at least a moderate likelihood of developing it in their lifetime. - Finally, 28% of respondents believed there was at least a moderate likelihood of DCIS spreading to other places in their body. At 18 months, mean perceived risk had not changed substantially (P=0.38). - Anxiety as measured by the Hospitalized Anxiety and Depression Scale (i.e., score ≥ 11) was the variable that was most consistently and strongly associated with heightened risk perceptions (perceived DCIS recurring within 5 years: OR=4.0, 95% CI=1.6 to 9.9, P=0.003; invasive breast cancer within 5 years: OR=4.3, 95% CI=1.9 to 9.9, P<0.001; and invasive breast cancer during lifetime: OR=5.3, 95% CI=2.0 to 14.3, P<0.001). |
| Partridge [59]  2008  USA | Questionnaire | To evaluate experiences, attitudes, and management approaches of a large sample of physicians who care for women with DCIS. | 208 physicians of patients with DCIS were sent the survey. 151 physicians (73%) completed the survey. | - Related to distress, 66% of physicians rated the emotional distress that women generally experience when diagnosed with DCIS as very high (4 or 5 on a 5 point scale). - Similarly, 64% of physicians perceived the treatment decision making process for women who have DCIS to be quite difficult (4 or 5 on a 5 point scale). However, physicians varied regarding their perceptions of the difficulty of the treatment decision making process for women who have DCIS in comparison with women who are diagnosed with invasive breast cancer. 78% of respondents indicated that the DCIS decision-making process was as or more difficult than that for invasive breast cancer (36% felt it was as difficult, 42% more difficult). - Regarding the general risk that DCIS poses to patients' overall long-term health, 64% indicated that it posed no or only a slight risk. - Physicians who care for proportionately fewer patients with breast cancer are 2.2 times more likely to view DCIS as a riskier disease to a patient's overall health compared with physicians for whom patients with breast cancer make up ≥ 40% of their practice (odds ratio, 2.2; 95% CI, 1.1-4.6; P=0.036). - Physicians were asked how they typically present a diagnosis of DCIS to their patients. 63% reported that they "always" or "almost always" refer to DCIS as cancer and 21% "never" or "almost never" refer to DCIS as cancer. - Physicians were also asked to indicate the 2 most common terms they use when discussing DCIS with patients. The majority of physicians selected "DCIS" or "non-invasive cancer" as their first (76%) or second (67%) choice. However, physicians selected many other choices and even wrote in a few that had not been supplied as response options in the survey. |
| Wong [60]  2008  Canada | Qualitative (interviews) | To examine Chinese-Canadian women's experience with (1) treatment decision making (mastectomy or breast-conserving surgery) and (2) their reflections on the treatment decision-making process. | Of the 46 women contacted, 37 self-identified themselves as having a Chinese ethnic background and therefore were eligible for the study. 26 agreed to participate for a response rate of 70%.  Mean age was 52.2 years. | *Women's understanding of their DCIS diagnosis:*   - Women most often referred to simply "having breast cancer" and only 5 women specifically referred to their cancer as DCIS, while 2 described it as a "precancer". - Although labeling their disease as "breast cancer" reinforced and significantly heightened fears and anxiety about dying prematurely among many participants, other were not as quick to jump to the conclusion that they had breast cancer. Some were confused about their diagnosis because DCIS was not explained in a way they could understand because they received inconsistent explanations. - No differences were evident between women who had breast-conserving surgery and those who had a mastectomy in their understanding of their DCIS diagnosis. - 5 women did understand their type of breast cancer was confined to the "milk ducts" and recalled that physicians drew pictures to help them understand their diagnosis. However, the explanations that were presented were inadequate. - Because of their uncertainties and difficulties accessing information they could understand, the women attempted to make sense of their diagnosis by drawing on the knowledge of friends and family members, and other breast cancer patients. - In the absence of a good understanding of DCIS, however, fears of reoccurrence and of suffering and premature death were predominant. - Years after their diagnosis and treatment for DCIS, some participants still had unanswered questions about their breast cancer and risk of reoccurrence.   *Women's experiences related to treatment decision making:*   - 4 women recalled their doctors simply indicated that mastectomy was the treatment for DCIS and did not engage them in any discussion of treatment options. - In one case, the woman reported that she was told she needed a mastectomy because the "spots" were widespread and too deep. - In the other case, the mastectomy was recommended by the specialist and primary care physician because of a family history of breast cancer. - 2 women received the recommendation to have a lumpectomy by a surgeon, and they followed this advice without asking questions. - In all other instances, the women recalled being presented treatment options, although some women's (n=13) resolve to have a mastectomy deterred them from seriously considering any other options. - 9 women appeared to have a good understanding of the disease and its treatments. Even though these women reported being more comfortable reading and speaking in either Cantonese or Mandarin, they spent considerable time and energy seeking additional information and deliberating about the treatment options. The women sought information in their own language through Chinese-speaking family physicians, library books, Chinese-speaking friends, and the toll-free Chinese Cancer Hotline. - Some women were frustrated that physicians did not give advice or provide information to help them understand the risk of reoccurrence associated with the different treatment options. Their reports suggested there was wide variation in the amount of information received about the treatments and the degree to which women were supported in treatment decision making. - Across all women, 3 subthemes describing women's treatment decision making were identified: getting rid of breast cancer once and for all, physician recommendations, and deliberation about treatment options.   *Reflection on the treatment decision-making process:*   - Regardless of their treatment, women emphasized how they had experienced a restored sense of satisfaction with their lives. Women discussed enjoying the present instead of living for the future. The experience of DCIS prompted some of the women to engage in healthy lifestyles, while others took on a "live for today" attitude. - With the worst behind them, however, 6 women questioned the information and recommendations received and the degree to which they were involved in the decision-making process. In reflecting on their experiences, several of the women wished that they had been more involved in making treatment decisions. Although most women who had a mastectomy downplayed the importance of the physical changes associated with a mastectomy, 3 talked about their difficulty accepting their changed body image. |
| Napoles-Springer [61]  2007  USA | Qualitative (interviews) | This study compared White women with Latina women’s understanding of their DCIS diagnosis, their treatment decision-making processes, and their satisfaction with care. | 18 White women and 16 Latina women participated in the study.  43% of Latina women were between ages 60-69 years and 33% of White women were ≥ 70 years  Out of all participants, over half were aged 60 or older (mean = 60.5 years), 69.7% were married, and 35.5% had a high school diploma or less, with non-Latina Whites more likely than Latinas to report a college education or higher. | - Four themes emerged from the interviews: patients’ understanding of their diagnosis, prognosis and treatment; emotional responses to the diagnosis; treatment decision-making processes; and factors associated with satisfaction with care. - Ethnic differences were observed in cognitive and emotional responses to DCIS, with White women generally reporting a better understanding of their diagnosis and treatment, and Latinas reporting more distress. - Regardless of ethnicity, women with DCIS preferred that physicians discuss treatment options and attend to their informational and emotional needs. - Satisfaction was associated with adequate information, expediency of care, and physicians’ sensitivity to patients’ emotional needs. - In response to a question asking for the meaning of the term “DCIS,” many women were unable to accurately state what the acronym represented. However, even if many did not know what the letters represented, most of the non-Latina White women were able to provide a description indicating a fairly accurate understanding of their diagnosis. - The ability to state a clear description of their diagnosis was the exception among Latinas. - Even Latinas who were fluent in English had problems verbalizing a clear-description of their diagnosis. Only 3/16 Latinas clearly stated that their condition was non-invasive vs. all 18 of the non-Latina White women stated that their condition was non-invasive. - Women in both groups clearly favored being presented with options for treatment - Most women (8 Whites, 7 Latinas) felt that treatment decision had been made jointly between themselves and their physicians. - For both Latina (6/16) and White (5/18) women, the most frequently cited factor affecting treatment decisions was the need to get rid of the disease to the greatest extent possible (“just get rid of it”). - Overall, the majority of women appeared satisfied with their treatment decisions and care. |
| Nekhlyudov [62]  2006  USA | Questionnaire | To measure changes in health-related quality-of-life in a large sample of women enrolled in two Nurses’ Health Study cohorts. | The analysis included 114,728 women who completed the health-related quality of life surveys between 1992 and 2000.  Of those, 510 were diagnosed with DCIS between 1992 and 2000. | - Women with DCIS were older (mean, 52.4 years; SD, 10.5 years) compared with those who remained free of DCIS (mean, 47.8 years; SD, 11.5 years). Health-related quality of life scores were obtained at three time points: 1992, 1996, and 2000. - Women with DCIS had statistically significantly greater declines in than those experienced by women without DCIS in the domains of role limitations due to physical problems (mean difference, -6.32 points; SE, 1.71), vitality (mean difference, -1.93; SE, 0.79), and social function (mean difference, -2.46; SE 0.94). However, these differences did not seem clinically significant. - Of the 204 women diagnosed during 1992 to 1996, 126 (61.8%) completed the health-related quality of life assessment in 2000. - Whereas declines after diagnosis occurred in role limitations due to physical problems, bodily pain, and social function, these were not present at the long term follow-up. For all domains, the long-term scores seemed almost identical to those without DCIS. - Among women with DCIS, short-term clinically important declines were more common among women diagnosed within 6 months of the follow-up survey than among those diagnosed more than 6 months before the survey, particularly in the domains of role limitations due to physical problems (42.7% v 30.0%), social function (30.5% v 21.1%), and mental health (21.1% v 10.5%). - Women more recently diagnosed were more likely to have clinically significant short-term declines in the domains of social functioning (OR, 1.78; 95% CI, 1.03 to 3.07) and mental health (OR, 2.03; 95% CI, 1.09 to 3.79). There were no association with treatment in these domains. - In comparison, among those who remained free of DCIS, the age-adjusted percentages of women who experienced at least a 10-point decline in the two clinically significant domains during the 4 year period was 20% for social functioning and 12% for mental health. |
| Prinjha [63]  2006  UK | Qualitative (interviews) | To explore attitudes towards screening mammography and information provision from the perspective of women with DCIS. | 10 respondents who had attended for routine screening mammography and were diagnosed with DCIS.  Ages ranged from 52-69. | - The respondents knew little about breast screening and cancer before their diagnosis, but were aware that breast cancer was common. - Women said they attended for screening because they had believed it was responsible health behaviour. All of them wanted to know that they were healthy and 'free of cancer' and six women said they had gained reassurance from being screened before. All 10 women said they knew little about the potential risks of screening mammography and felt that because screening was so strongly recommended by health professionals, government, and the media, it must be a good thing. - While women tend to associate breast lumps, and occasionally other symptoms with breast cancer, none had heard of DCIS before being screened. - When diagnosed with it, all 10 women were confused as to what it was and whether it was cancerous. Five women believed they had invasive breast cancer. - Terms such as a pre-cancerous, pre-invasive, non-invasive, intraductal or non-progressive cancer left women feeling confused. - Women searched for information about DCIS at different stages: between diagnosis and surgery, shortly after surgery, during and after recovery. They obtained information from various sources, including the Internet, books, leaflets, medical journals, cancer charities, and health professionals. Some women are wary of finding out too much, or being alarmed by distressing statistics. However, those who sought information sometimes found it inadequate and confusing because of the uncertain natural progression of the condition and the still-emerging evidence about the effectiveness of various treatment options. - Those women who sought out information before having surgery wanted to know more about how serious their DCIS was, whether it was likely to progress and whether surgery was really necessary, and did not receive clear information from their doctors. - 4 women sought information about DCIS and their own case only after surgery. They felt they had been rushed into surgery and wondered by a pre-cancerous condition should be treated so urgently. - 4 women wondered whether they would have opted for surgery at all had they known DCIS has an uncertain natural progression and theirs might have never progressed. - 6 women were shocked to discover the large numbers of women diagnosed with DCIS annually and questioned why the NHS breast-screening leaflet they had received made no mention of it. They felt that this omission, along with the omission of information about the potential harms of screening mammography, prevented them from making fully informed decisions about whether to attend. In retrospect, these women said they would have liked more information about mammographic screening before attending. - Having been diagnosed with DCIS and learned more about it and mammographic screening generally, women now felt more able to make an informed choice about whether to have mammograms in the future. - Having learned how poorly understood DCIS was, 3 of these women regretted ever having gone for screening and doubted whether it should be offered for a condition that doctors do not know how to treat and which could remain harmless for many years. - 4 women felt that if they were to be diagnosed with DCIS again, they might defer surgery or refuse to have it at all. - 6 women chose to continue to have mammograms. 4 of them said they found follow-up appointments reassuring. 3 women were glad they had been screened, believing that it had prevented them from developing invasive cancer and saved their lives, and therefore encouraged other women to attend. |
| van Gestel [64]  2006  Netherlands | Questionnaire | To compare the health-related quality of life, impact of the disease, risk perception of recurrence and dying of breast cancer, and understanding of diagnosis of patients with DCIS and invasive breast cancer 2-3 years after treatment. | Of the 180 patients who received the questionnaire, 135 patients returned a completed questionnaire (75% response).  Mean age at the time of completing the questionnaire was 61 years for the women with DCIS (N=33) and 59 years for the women with invasive breast cancer (N=91). | - Women with invasive breast cancer were more likely to have had breast-conserving therapy (P<0.001), sentinel node biopsy (P<0.001), radiotherapy (P<0.001) and chemotherapy (P=0.01) or hormonal therapy (P=0.04). - Women with DCIS had a significantly better score on the subscales bodily pain (85.4 versus 75.2, P=0.02) and general mental health (77.8 versus 70.5, P=0.05) compared to women with invasive breast cancer. - The most positive influence of the disease was seen on family relations, relationships with relatives, self-expression and the outlook on life. Women with DCIS experienced a more positive or less negative effect on their physical health (P<0.001), sex life (P=0.03) and relationships with friends/acquaintances (P=0.01) than women with invasive breast cancer. - Comparison of the mean numbers of negative, neutral and positive scored items revealed that patients with invasive cancer reported significantly more negative effects than those with DCIS. - For both groups, the reported number of positive effects was significantly greater than the number of negative effects (P<0.001). - Women with DCIS and invasive breast cancer reported comparable risk perceptions concerning the risk of recurrence and dying of their disease. In contrast, women with invasive breast cancer felt it more likely that the medical treatment was doing them more harm than good (P=0.01). - When asking women to describe their disease in an open question, women with DCIS often reported the term calcifications, whereas women with invasive breast cancer frequently described their disease as a (malignant) tumour. - Women with DCIS were significantly more accurate in stating their diagnosis compared to women with invasive breast cancer (P=0.02). Of the women with DCIS, 56% stated their diagnosis correctly, by marking the correct illustration, compared to only 21% of the women with invasive breast cancer. Approximately one third of the women with invasive breast cancer marked the illustration which represented DCIS. |
| Katz [65]  2005  USA | Questionnaire | To address patterns and correlated of local therapy. | Women aged 79 years and younger diagnosed with DCIS from Dec 2001 to Jan 2003.  659 patients who had DCIS were the sample used in this study.  Mean age was 58.8 years. | - 67.6% of patients were white, 19% were African-American, and 10.7% were other race. - Only 14% of patients at lowest risk of recurrence (based on tumor size and histologic grade) received a mastectomy compared with 22.8% and 52.6% of patients at intermediate and highest risk (p<0.001). - Age was also independently associated with surgical treatment because younger women were more likely than older women to receive mastectomy (odds ratios = 0.77, 0.49, and 0.28 for age groups 50 to 59, 60 to 69, 70 to 79 years, respectively). - Overall, 69% of patients reported that their surgeon(s) discussed both mastectomy and breast-conserving surgery, 26.7% reported that their surgeon(s) only described breast-conserving surgery, and 4.3% reported that their surgeon(s) only described mastectomy. - Overall, 31.9% of patients reported that their surgeon(s) did not recommend one procedure over the other, 52.8% reported that their surgeon recommended breast-conserving surgery, and 15.3% reported that their surgeon recommended mastectomy. - Only 13.1% of patients who were not influenced or slightly influenced by concerns about recurrence received mastectomy compared with 48.8% of women who were greatly influenced by this concern (p<0.001). - A between-geographic site difference in receipt of radiation after breast-conserving surgery was observed for the lowest risk group (38.9% in Los Angeles, 70.5% in Detroit) but not for the highest risk group (80.2% in Los Angeles, 85.9% in Detroit, p=0.006 for site and risk group differences). - Between-site differences in receipt of radiation after breast-conserving surgery were consistent with patient’s recall of surgeon discussions about treatment. |
| Rakovitch [66]  2003  Canada | Questionnaire | To assess how women with DCIS perceive their risks of recurrence, dying from breast cancer, and psychological distress compared to women with early stage invasive breast cancer. | 240 patients with a diagnosis of breast cancer were eligible for the study. 228 patients completed the survey (64 with DCIS; 164 with early stage invasive breast cancer).  The median age was 55 for DCIS patients and 57 for early stage invasive breast cancer patients. | - Women with DCIS were significantly better at accurately stating their diagnosis compared to women with early stage invasive breast cancer (43/64 (66%) vs. 51/164 (31%), P<0.001) and more women with DCIS were able to identify the correct diagram (42/64 (66%) vs. 37/164 (23%) respectively, P<0.001). - Overall, 37/64 (58%) of women with DCIS answered both questions correctly compared to 37/164 (23%) women with early stage invasive breast cancer (P<0.001). - Despite the finding that women with DCIS were better able to state their diagnosis, there were no significant differences in perceptions of risk related to the development of local recurrence or distant recurrence. Just as many women with DCIS as with early stage invasive breast cancer felt they were likely to develop a local recurrence (53% vs. 45%, P=0.14), and just as many women with DCIS as with early stage invasive breast cancer felt this was very likely to occur (13% vs. 12%, P=0.38). When asked about the risk of developing distant recurrence, the majority of women in both groups felt they were unlikely to develop distant metastases (64% vs. 61%, P=0.35). However, a similar proportion of women with DCIS and early stage invasive breast cancer felt they were likely (36% vs. 39%, P=0.35) and very likely (7% vs. 13%, P=0.10) to develop distant metastases. - The two groups also responded similarly when asked about their risk of breast cancer related mortality. About a quarter of each group (27% vs. 27%, P=0.50) felt they were likely to die of breast cancer, although more women with early stage invasive breast cancer felt they were very likely to die of breast cancer (3% vs. 13%, P=0.01). - Women with DCIS and early stage invasive breast cancer expressed similar levels of psychological distress related to their breast cancer. The most frequent and/or severe psychological symptoms were trouble sleeping (DCIS: 24% vs. early stage invasive breast cancer: 31%, P=0.15), unhappiness/depression (DCIS: 13% vs. early stage invasive breast cancer, 22%, P=0.06) and nervousness/anxiety (DCIS: 23% vs. early stage invasive breast cancer: 26%, P=0.29). - Women with DCIS reported a slightly smaller tendency towards withdrawing from close family/friends than women with early stage invasive breast cancer (5% vs. 11%, P=0.08), and less strain on interpersonal relationships (0 vs. 6%, P=0.02). |
| Shugg [67]  2002  Tasmania | Questionnaire | To describe the management of DCIS in 1995 in Australia. | 205 surgeons returned questionnaires for 418 incident cases. | - Surgeons were asked to indicate what investigations were ordered for the patient prior to surgery. There was an average of two investigations per case. Surgeons who saw 6-11 cases in the 6 month period did an average of two-investigations per case, while those who saw only one case of DCIS in the study period averaged 3 investigations per case. - The most common investigation was a diagnostic mammogram (49% of cases), while ultrasound was used in 25% of cases. - Surgeons treating fewer DCIS cases were more likely to seek a second opinion than were the more active surgeons (P<0.01). - Surgeons treating six or more cases of DCIS were most likely to perform breast conserving therapy, while surgeons treating only one DCIS case every 6 months were least likely to perform breast conserving therapy (P<0.05). - Women seeing surgeons working in non-urban centres were more likely to decline breast reconstruction after mastectomy than women seeing surgeons in urban centres (P<0.05). - Surgeons with higher caseloads were more likely to do breast reconstruction at the time of surgery than were surgeons seeing only one case every 6 months (P<0.01). - Women seen by a surgeon treating 6-11 DCIS cases were more likely to receive breast conserving therapy than were women treated by a surgeon treating only one DCIS case (OR=3.24, 95% CI: 1.32-7.96). - Clinicians' reasons for choosing mastectomy varied by caseload and to a lesser degree by the urban or non-urban status of the surgeon. - Concern about recurrence after breast conserving therapy and small size of the breast were indicators for mastectomy among surgeons treating fewer cases of DCIS (P<0.01 and P<0.01). - Concern about recurrence was also cited more frequently by non-urban (61%) than urban (25%) surgeons (P<0.01). - Caseload was related to surgeons reporting that they performed breast conserving therapy because their patient was keen to preserve the breast (P<0.05). - Surgeons treating six or more cases of DCIS were less likely to give this reason than those treating 2-5 cases of DCIS. - Of the 52 (13%) cases having axillary dissection, suspicion of invasive disease (40%) was the most frequently cited reason for this procedure. - The following reasons were given for performing axillary surgery on patients treated by mastectomy: suspicion of invasive disease (51%), routine practice (30%), size of tumour (27%), grade of tumour (24%), and tumour in axillary tail (11%). - Surgeons who did not refer their breast conserving therapy patients to a radiation oncologist were asked their reasons for this decision. Tumour size and patient's age were associated linearly with referral (P<0.05 and P<0.01, respectively). Surgeons were less likely to refer their older patients (e.g. women over 70) for radiotherapy. |
| Staradub [68]  2002  USA | Comparative cohort | To identify differences in demographic factors among women choosing breast conserving therapy, mastectomy, and mastectomy with immediate reconstruction who were offered a choice between the three procedures. | Of the 586 tumors in 578 women in this study, 108 tumors (18%) were DCIS, and 478 tumors (82%) were clinical Stage I or II invasive breast carcinoma.  The mean age of patients who underwent breast conserving therapy was 54 years, compared with a mean age of 59 years among patients undergoing mastectomy alone and 48 years among patients undergoing mastectomy with immediate reconstruction. | - Patients who chose breast conserving therapy were significantly younger compared with patients who opted for mastectomy alone (P<0.001), and patients who elected to undergo mastectomy with immediate reconstruction were significantly younger compared with patients who underwent either breast conserving therapy breast conserving therapy or mastectomy alone (P<0.001). - The stage distribution of patients who underwent breast conserving therapy differed significantly compared with the stage distribution of patients who underwent mastectomy alone (P<0.001). - Patients who underwent mastectomy alone were more likely to have a clinical Stage II breast carcinoma compared with patients who underwent breast conserving therapy (48% vs 31%, respectively; P<0.001). - Women who underwent mastectomy alone or mastectomy with immediate reconstruction were more likely than women who underwent breast conserving therapy to have had a breast biopsy in the past for benign disease (33% and 34% for mastectomy and mastectomy with immediate reconstruction vs. 20% for breast conserving therapy; P=0.02). - The insurance status of patients who underwent mastectomy with immediate reconstruction differed significantly compared with patients who underwent breast conserving therapy or mastectomy alone. The group that underwent mastectomy with immediate reconstruction was significantly more likely to have private or preferred provider organization insurance compared with the group that underwent mastectomy alone (88% vs. 60%, respectively; P=0.02). |
| De Morgan [69]  2002  Australia | Qualitative study  (focus groups) | To explore women's experience of being diagnosed with DCIS in relation to the following: response to the diagnosis; understanding about the diagnosis; satisfaction with information; satisfaction with the level of involvement in treatment decision-making and satisfaction with support services. | 26 women diagnosed with DCIS in 5 focus groups. | - The study found that women were confused about whether or not they had cancer that could result in death. - Women's confusion was compounded by the use of the term 'carcinoma' and by the recommendation of treatments such as mastectomy. - Women's confusion was not alleviated by appropriate information, with most women reporting dissatisfaction with the information they received specifically about DCIS. |
| Bluman [70]  2001  USA | Questionnaire | To explore a range of psychosocial, behavioral, and educational issues in women who had been diagnosed with DCIS. | 122 women were contacted, 76 completed questionnaires.  The mean age of the study participants was 56 year’s 91% of the women were white, 72% were married or living as married, and 59% attended at least some college. | - Most women were satisfied with several aspects of their care since being diagnosed with DCIS. - 37% were not satisfied or only somewhat satisfied with information about future health problems related to DCIS and 15% were not satisfied with support they received from the doctor. - 61% of women rated a cancer recurrence within the next 5 years as either moderately likely, likely or very likely. - Lower levels of satisfaction with treatment choice and treatment information were associated with higher levels of worry about breast cancer recurrence at high statistically significant levels (p=0.01). - Women had considered or done the following to reduce cancer risk since their DCIS diagnoses: exercising (66%), making dietary changes (65%), not taking hormone replacement therapy (59%), taking vitamins (58%), and participating in a tamoxifen study (16%). - Among the women who considered themselves to be sexually active, 50% reported decreased interest in sex and decreased sexual activity since their cancer diagnoses. - 33% reported feelings of sexual unattractiveness, and 19% experienced pain or difficulty with intercourse. |
| Brown [71]  2000  Australia | Qualitative (interviews) | To gain a better understanding of the experience of women who have had surgery for non-invasive breast cancer and issues surrounding information. | 6 women who had undergone surgery for DCIS, half of the women had partners living with them. | - The findings highlight the individual nature of the experience of information with the women wanting information that is tailored to their personal needs. - Nurses can provide care that is more responsive to the information needs of each woman by being aware of the diverse ways in which women experience information. - For all women, the surgeon was a very important communicator of information, although the majority use other sources (such as the Internet) of information to supplement this. - Overall, information was one of the most important means of navigating the experience of being diagnosed and treated for DCIS. |
| Amichetti [72]  1999  Italy | Questionnaire | To evaluate the quality of life in patients with DCIS of the breast treated with conservative surgery and postoperative irradiation. | 106 female DCIS patients treated between 1980-1990.  Median age at time of treatment was 50 and median age at time of questionnaire was 54 years.  36 patients had primary school education; 33 were homemakers. | - The questionnaire was completed by 83 patients (78%), who had a median follow-up of 54.5 months. The patients claimed to be in good physical condition. - Data relating to sexual life were provided by 93% of the sample. - Some limitations in sexuality, some interference with sexual desire, and some modifications during intercourse were reported by 5, 6, and 5 patients, respectively. - The subjective evaluations of the cosmetic results of the therapies were generally good. Only 13 patients (16%) reported the perception of a worsened body image. - 46% of the sample (38 patients) declared that they felt tense, 48% (39 patients) nervous, 29% (38 patients) lonely, 59% (41 patients) anxious, and 41% (34 patients) depressed. - Only seven patients (8%) declared that the treatment had had a bad effect on their social life, and 15 (18%) thought that their current life had been affected by the treatment. The amount of information received concerning the disease and treatment (surgery and radiotherapy) was considered sufficient by 79%, 75%, and 79% of the sample, respectively. |
| Webb [73]  1997  UK | Interviews | To explore women's experience of DCIS, in an attempt to begin exploration of possible similarities or differences in the way women experience invasive and non-invasive breast cancer. | Mean age of the sample was 66 (range 55-81).  The study was limited to 10 participants. | - Women whose DCIS had been found on routine screening tended to reply that they had not discovered the problem themselves. - Most commonly, they had received a telephone call from the mammography clinic asking them to return for "more tests". The majority of women had received the first news of a potential problem by telephone but it was not clear who had made this call. - The principal reaction to the diagnosis was a calm acceptance of something about which they could do nothing about, and therefore, not worth getting upset over. Only three women reported being upset initially, but for two of them this was short-lived. - Information given to the women seems to have been deficient in one hospital. In another hospital however, patients seemed more satisfied with the information they had been given. - None of the women appeared to know that they had a non-invasive form of cancer. |
